# From Field to Extract: Controlling Post-Harvest and Extraction Variables for Reproducible Recovery of Cannabinoids and Terpenes from *Cannabis sativa* L. by Sequential Hydrodistillation and Pressurized Liquid Extraction

**DOI:** 10.3390/molecules31213955

**Published:** 2026-11-02

**Authors:** Carla Hamann, Olivia Jansen, Lucas Teilliez, Pierre-Yves Sacré, Kateline Jullien, Françoise Bafort, Erik Maquoi, Allison Ledoux, Michel Frédérich

**Affiliations:** 1 Laboratory of Pharmacognosy, Center for Interdisciplinary Research on Medicines, University of Liège, 4000 Liège, Belgium; chamann@uliege.be (C.H.); ojansen@uliege.be (O.J.); lucasteilliez@gmail.com (L.T.); kateline.jullien@uliege.be (K.J.); erik.maquoi@uliege.be (E.M.); allison.ledoux@uliege.be (A.L.); 2 Research Support Unit in Chemometrics, Center for Interdisciplinary Research on Medicines, University of Liège, 4000 Liège, Belgium; pysacre@uliege.be; 3 Integrated and Urban Plant Pathology Laboratory, Gembloux Agro-Bio Tech, University of Liège, 5030 Gembloux, Belgium; francoise.bafort@uliege.be; 4 Laboratory of Tumor and Development Biology, GIGA Institute, University of Liège, 4000 Liège, Belgium

**Keywords:** cannabinoids, decarboxylation, entourage effect, terpenes, freezing, hemp

## Abstract

Valorizing the full phytochemical profile of hemp (*Cannabis sativa* L.), including the synergistic activity often referred to as the “entourage effect,” requires efficient recovery of both its cannabinoid and terpene fractions. However, initiating cannabinoid decarboxylation through high drying temperatures leads to substantial loss of the terpene profile, making the two objectives thermally incompatible in conventional workflows. This study first investigated the influence of post-harvest conditions on cannabinoid content and decarboxylation using a Design of Experiments approach, and then developed a process bypassing the drying step, based on freezing and hydrodistillation, to formulate a broad-spectrum product combining high cannabinoid and terpene content. Two hemp chemotypes (Felina 32, CBD-dominant; Santhica 27, CBG-dominant) were characterized, and drying temperature was identified as the critical parameter governing decarboxylation. Freezing before hydrodistillation preserved monoterpenes and improved essential oil yield relative to oven-drying, while the heat of hydrodistillation itself drove in situ cannabinoid decarboxylation, enabling near-complete recovery in a subsequent pressurized liquid extraction step. This integrated process achieved near-quantitative recovery of cannabinoids and monoterpenes across independent production batches, with encouraging recovery of sesquiterpenes, supporting the standardized and reproducible valorization of hemp, especially for pharmaceutical applications.

## 1. Introduction

*Cannabis sativa* L. owes much of its pharmacological and industrial interest to two classes of specialized metabolites: the phytocannabinoids and the terpenes [1,2]. Considerable evidence suggests that the biological activity of cannabis preparations reflects not the action of single molecules but the combined contribution of multiple constituents, known as the “entourage effect” [3,4,5,6]. Under this hypothesis, terpenes and minor cannabinoids can modulate the activity of the major ones, and complex botanical preparations could outperform equivalent doses of isolated compounds [7,8,9]. Exploiting this potential synergy requires an extract that retains both classes in their desired forms: cannabinoids in their decarboxylated (neutral) state, and terpenes, especially the volatile monoterpenes, preserved intact. Producing such an extract reproducibly, from a single biomass, is the problem addressed here.

Much of the published data on cannabis extracts is difficult to compare across studies because the phytochemical composition and quality of the starting material are poorly controlled and, crucially, its post-harvest treatments are rarely reported [10].

Several post-harvest factors can influence the cannabinoid and terpene composition of the biomass, including drying temperature, handling, grinding, and storage conditions [11,12]. Various drying technologies have been investigated for cannabis post-harvest processing, including traditional hang drying (air drying), oven drying, freeze-drying, and microwave-assisted drying [12,13,14]. While oven drying is widely used for its simplicity and low cost, it presents several limitations, including terpene volatilization, premature cannabinoid decarboxylation, heterogeneous moisture distribution, and uneven drying rates, collectively compromising phytochemical integrity and batch-to-batch reproducibility [15,16]. Freeze-drying better preserves the terpene profile but is costly and less accessible at industrial scale [14]. Because post-harvest conditions are rarely documented in the literature, comparable extracts may in fact derive from chemically different materials. In 2024, the European Pharmacopoeia introduced a dedicated monograph for cannabis flower, underlining the need to control the raw material quality [17]. Reproducible cannabis extracts therefore require quality control that begins in the field, before any extraction.

On the extraction side, the three primary commercial approaches include solvent-based extraction (commonly ethanol), supercritical CO_2_ extraction, and hydrodistillation, each yielding distinct cannabinoid and terpene profiles [13,18]. Hydrodistillation produces the richest terpene fraction, supercritical CO_2_ maximizes cannabinoid yield, and ethanol extraction predominantly retains acid cannabinoid forms with lower terpene recovery [18].

We first set out to optimize a conventional drying and extraction process for the recovery of decarboxylated cannabinoids, using a Design of Experiments (DoE) across two chemotypes. We hypothesized that this might reveal a fundamental constraint: if cannabinoid decarboxylation is governed largely by the heat applied during drying rather than during extraction, then the same heat could simultaneously volatilize the terpene fraction intended for preservation, such that the two objectives might not be reconcilable within a single temperature setting. Under this hypothesis, terpene preservation and cannabinoid decarboxylation would need to be decoupled, in time and by fraction, rather than reconciled in a single step.

Other authors have already highlighted the value of in situ cannabinoid decarboxylation during distillation-based processes: microwave-assisted hydrodistillation of Cannabis inflorescences was reported to drive substantial phytocannabinoid decarboxylation (approximately 70%) while recovering the terpene fraction [19], and conventional steam distillation of CBD-hemp biomass was similarly shown to yield both an essential oil and a decarboxylated residue [20]. These studies indicate that distillation heat alone can drive cannabinoid decarboxylation without necessarily degrading the biomass, but they remain proof-of-concept observations on a single batch, using microwave assistance or steam distillation.

Building on this rationale, two design choices structure the integrated process developed in the present work. First, fresh biomass is frozen immediately after harvest instead of being thermally dried, in order to protect the volatile monoterpenes and remove drying as an uncontrolled source of variability. Second, the heat required for cannabinoid decarboxylation is no longer supplied by drying but by hydrodistillation itself, a single step intended to recover the terpene fraction as an essential oil while simultaneously promoting decarboxylation within the biomass. The resulting, largely decarboxylated residual plant biomass could then be extracted with ethanol to recover the remaining cannabinoids, offering the possibility of combining both fractions from a single plant into extracts of tailored composition.

We evaluated this integrated process across two chemotypes harvested within a single season, distinct from the seasons used for the DoE trials, and further assessed it the following year on an additional variety, allowing reproducibility to be tested not only between chemotypes but between growing seasons of genuinely different raw material, a source of variability rarely addressed in extraction studies. Cannabidiol (CBD) and cannabigerol (CBG) were selected as the primary analytical targets, as they represent the dominant neutral cannabinoids in the two hemp chemotypes investigated (the CBD-dominant Felina 32 and the CBG-dominant Santhica 27), as characterized in the present study. Their respective acid precursors, cannabidiolic acid (CBDA) and cannabigerolic acid (CBGA), were monitored in parallel as markers of decarboxylation status. Other cannabinoids present at trace levels, including tetrahydrocannabinol (THC) and its acid precursor tetrahydrocannabinolic acid (THCA) (≤0.3% *w*/*w*, in compliance with EU regulations), were outside the scope of this study.

We aimed to determine whether quantifying the influence of post-harvest conditions and then controlling each subsequent step from extraction through recombination could resolve this apparently intractable trade-off and yield reproducible, standardizable extracts of tailored composition.

## 2. Results

### 2.1. Preliminary Observations: Incomplete Decarboxylation Under Low-Temperature Drying

In natural product research, characterizing the phytochemical quality of the harvested material before any extraction step is essential. Accordingly, our workflow systematically includes quantification of both the acidic and neutral forms of cannabinoids in the dried raw material from each harvest.

**Figure 1:**
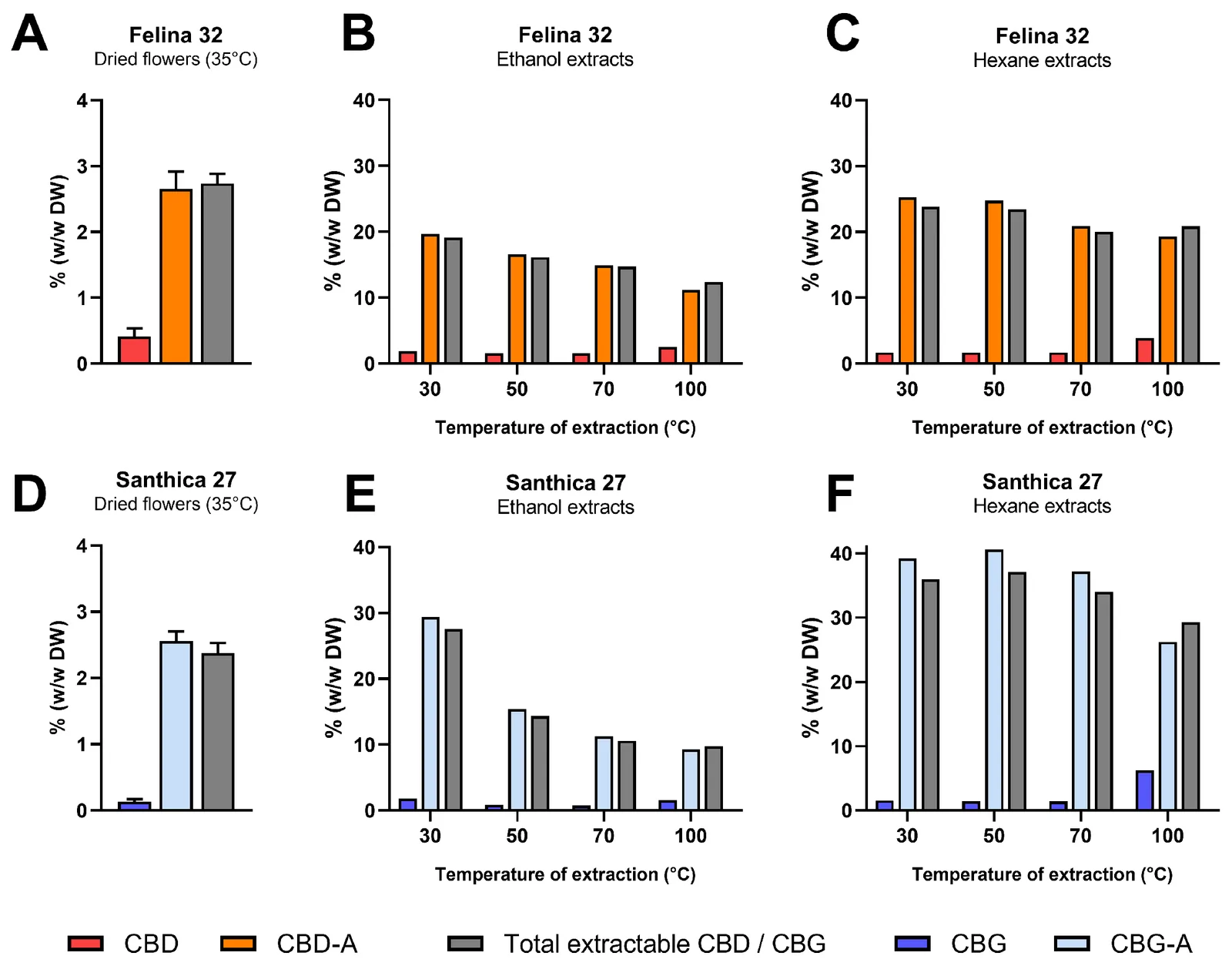
Cannabinoid quantification of Felina 32 and Santhica 27 hemp flowers and derived extracts from the 2022 harvest. (**A**,**D**) CBD, CBDA and CBG, CBGA content (% *w*/*w* DW) in raw plant material dried at 35 ± 5 °C in a ventilated oven. Cannabinoid profile of ethanol extracts (**B**,**E**) and hexane extracts (**C**,**F**) from Felina 32 and Santhica 27 dried flowers at increasing extraction temperatures (30, 50, 70, and 100 °C). Data represent mean ± SD (n = 4) for raw material and single measurements for extracts. DW: dry weight.

For the 2022 harvest, Felina 32 and Santhica 27 flowers were dried at low temperature (35 ± 5 °C) and cannabinoids remained predominantly in their acidic form, with cannabidiolic acid (CBDA) and cannabigerolic acid (CBGA) accounting for the majority of the total cannabinoid content in the raw material (Figure 1A,D). To evaluate whether decarboxylation could instead be achieved during extraction, ethanol and hexane extracts were prepared at increasing extraction temperatures (30, 50, 70, and 100 °C). Across this temperature range, neither solvent yielded fully decarboxylated extracts: CBDA and CBGA remained detectable even at the highest extraction temperature tested in both Felina 32 (Figure 1B,C) and Santhica 27 (Figure 1E,F).

These observations suggested that drying conditions, rather than extraction conditions, seem to govern the extent of cannabinoid decarboxylation, a hypothesis further investigated through a Design of Experiments approach, presented in the following section.

### 2.2. DoE Identifies Drying Temperature as the Critical Parameter

**Figure 2:**
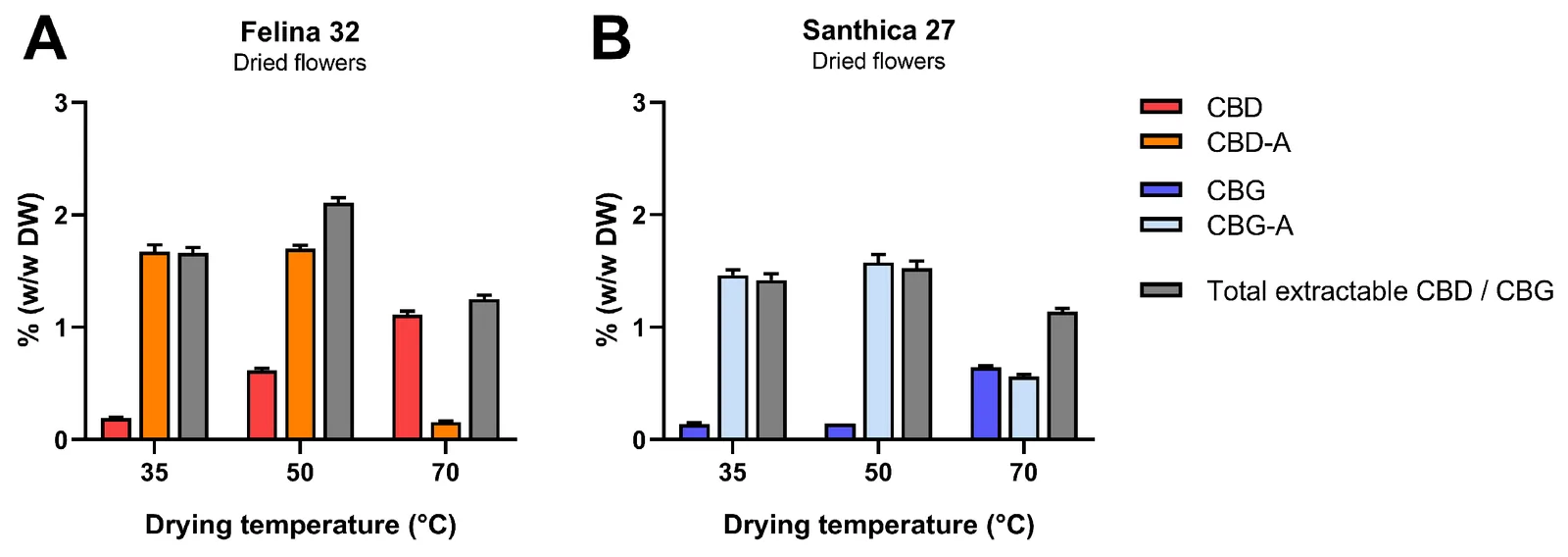
Acidic and neutral cannabinoid content of Felina 32 and Santhica 27 raw material at three drying temperatures. (**A**) CBD, CBDA and (**B**) CBG, CBGA content (% *w*/*w* DW) in raw plant material dried at 35, 50, or 70 °C in a ventilated oven. Raw material from the 2023 harvest, used for the design of experiments. Data represent mean ± SD (n = 3). DW: dry weight.

For the purpose of the DoE, the 2023 harvest was divided into three groups, dried at 35, 50, or 70 °C in a ventilated oven. Prior to DoE analysis, the phytochemical composition of each raw material group was assessed by HPLC-DAD to quantify acidic and neutral forms of cannabinoids. In Felina 32, neutral CBD content increased with drying temperature, from 0.2% (*w*/*w* DW) at 35 °C to 1.1% at 70 °C. Expressed as a proportion of total CBD, the decarboxylated fraction increased from 12% at 35 °C to 89% at 70 °C, indicating that decarboxylation becomes substantial only above 50 °C (Figure 2A). A similar but less pronounced trend was observed for CBG in Santhica 27. The decarboxylated fraction rose from 9% at 35 °C to 56% at 70 °C (Figure 2B), indicating a lower decarboxylation efficiency than CBD under the same conditions.

**Figure 3:**
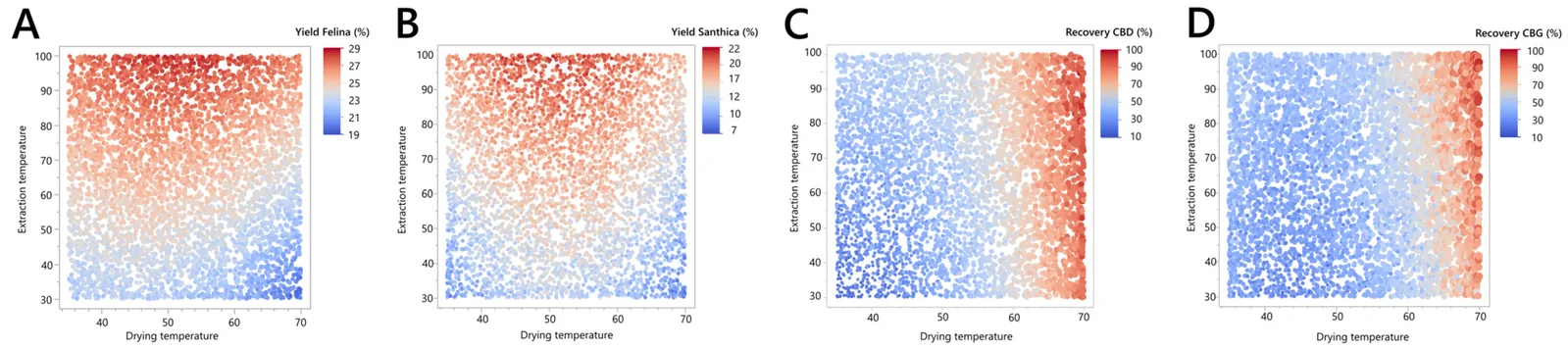
Predicted extraction yield in Felina 32 (**A**) and Santhica 27 (**B**), and predicted cannabinoid recovery in Felina 32 (**C**) and Santhica 27 (**D**), obtained by Monte Carlo simulation (5000 runs) of the reduced models. Drying and extraction temperature, the two most influential factors identified by the reduced models, are shown on the axes. Cycle number and cycle duration, which had smaller effects, were also varied within their tested ranges in the simulation but are not represented on the axes.

Reduced models were fitted for each response, retaining the most influential terms together with the main effects required by model hierarchy. The full set of 24 runs is provided in Supplementary Tables S1 and S2 (Felina 32 and Santhica 27). Model fit statistics (PRESS R^2^ and RMSE) for each reduced model are given in Appendix A, Table A2. Extraction yield was mainly governed by extraction temperature in both varieties (Figure 3A,B). In Felina 32, drying temperature had only a slight effect on yield. In Santhica 27, drying temperature had a concave effect on yield, with a maximum around 50 °C and lower yields at 35 and 70 °C. Cycle duration had a smaller positive effect.

Drying temperature was the dominant factor for cannabinoid recovery in both varieties. In Felina 32, CBD recovery increased slowly between 35 and about 50 °C, then steeply up to 70 °C, indicating a convex response (Figure 3C).

In Santhica 27, CBG recovery remained low up to about 50 °C before increasing markedly but reached lower values than CBD recovery in Felina 32 under comparable conditions (Figure 3D). Extraction temperature had a smaller positive effect on both recoveries.

Collectively, these results highlight drying temperature as the critical determinant of cannabinoid decarboxylation, with substantial decarboxylation only observed above 50 °C and most efficiently at 70 °C. To monitor terpene integrity across DoE conditions, β-caryophyllene (BCP) was used as a marker of the sesquiterpene fraction. Myrcene (MYR), used later as a monoterpene marker, fell below the detection limit in these samples. BCP levels were markedly reduced in plant material dried above 50 °C (Supplementary Tables S1 and S2, for Felina 32 and Santhica 27, respectively), indicating that no drying condition simultaneously achieved cannabinoid decarboxylation and terpene preservation within the oven-drying framework.

Clearly, drying hemp flowers in an oven leads to significant alterations in phytochemical composition and does not represent a suitable approach for producing concentrated, reproducible, and terpene-rich extracts. These findings prompted the development of an alternative process, based on freezing followed by hydrodistillation, designed to simultaneously preserve terpene integrity and achieve controlled cannabinoid decarboxylation.

### 2.3. From a Single Plant Batch to a Full-Spectrum, High-Concentration Product

Starting with the 2024 harvest, fresh hemp inflorescences were frozen immediately after harvest rather than oven-dried. As described in Section 4.4, a representative subsample of each batch was oven-dried to determine the fresh-to-dry weight ratio and to characterize the raw material. HPLC analyses determined a total extractable CBD content of 3.29% (*w*/*w* DW) in Felina 32 and a total extractable CBG of 1.94% (*w*/*w* DW) in Santhica 27, with a fresh-to-dry weight loss of 62.5% and 57.9%, respectively.

**Figure 4:**
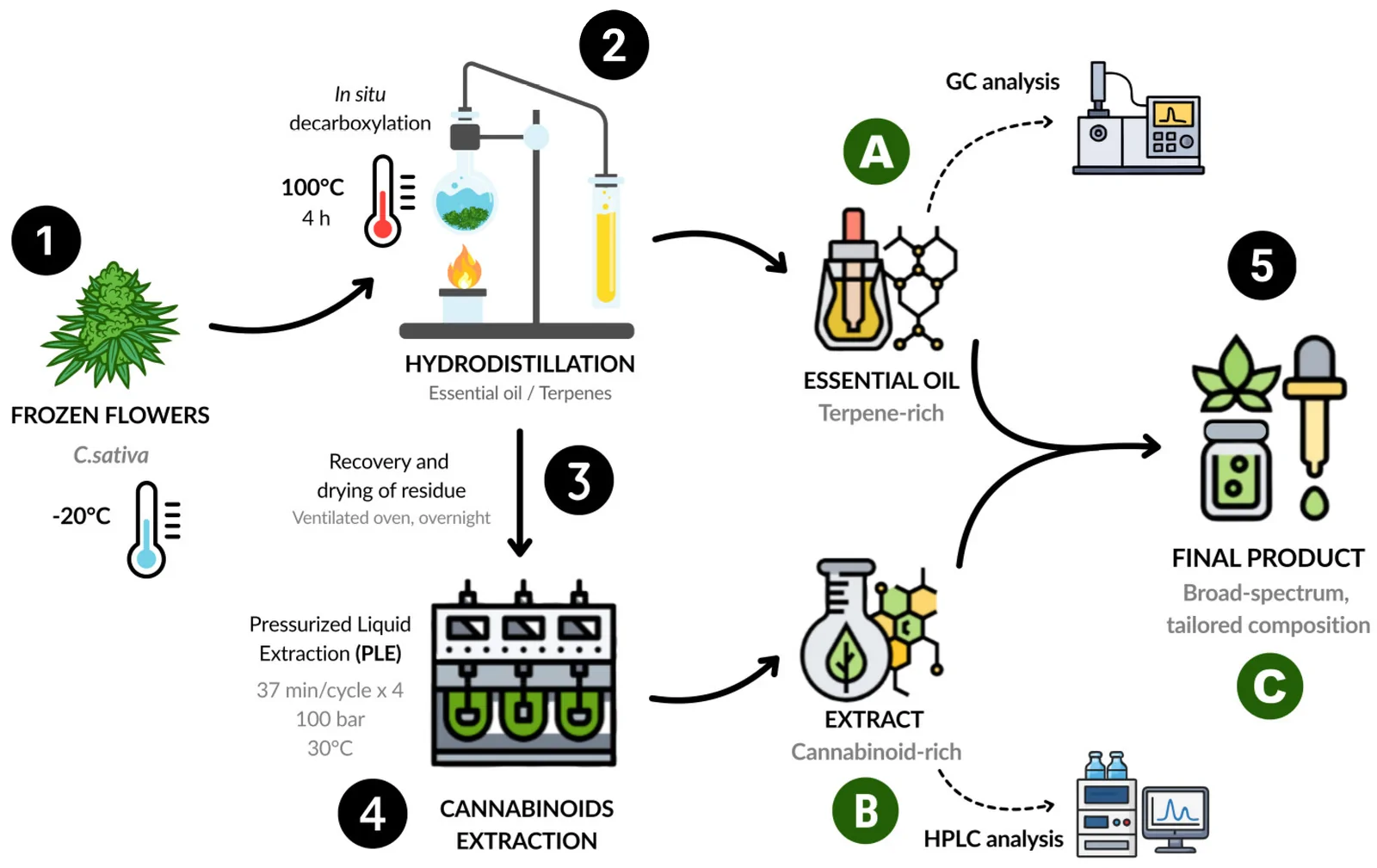
Integrated process for the extraction of cannabinoids and terpenes from *Cannabis sativa* L. plant material. Frozen inflorescences of *C. sativa* (**1**) are subjected to hydrodistillation in boiling water (100 °C, 4 h), which recovers the volatile terpene fraction while promoting in situ decarboxylation of the cannabinoids (**2**). The solid residue is recovered, dried and ground (**3**), then submitted to pressurized liquid extraction (PLE; 4 cycles of 37 min, 100 bar, 30 °C) to recover the cannabinoid fraction (**4**). The two fractions obtained, a terpene-rich essential oil (**A**) and a cannabinoid-rich extract (**B**), are characterized by GC and HPLC, respectively, and recombined at defined ratios (**5**) to yield a broad-spectrum final product of tailored composition (**C**).

The integrated process developed in this study is summarized in Figure 4: frozen inflorescences are subjected to hydrodistillation (HD), which allows us to recover the volatile terpene fraction as an essential oil while simultaneously promoting in situ cannabinoid decarboxylation within the residual biomass. The residue is then dried, ground, and submitted to pressurized liquid extraction (PLE) with ethanol as solvent to recover the cannabinoid fraction. The two resulting fractions, essential oil and cannabinoid-rich extract, can be recombined at selected ratios to yield a broad-spectrum product of tailored composition.

**Figure 5:**
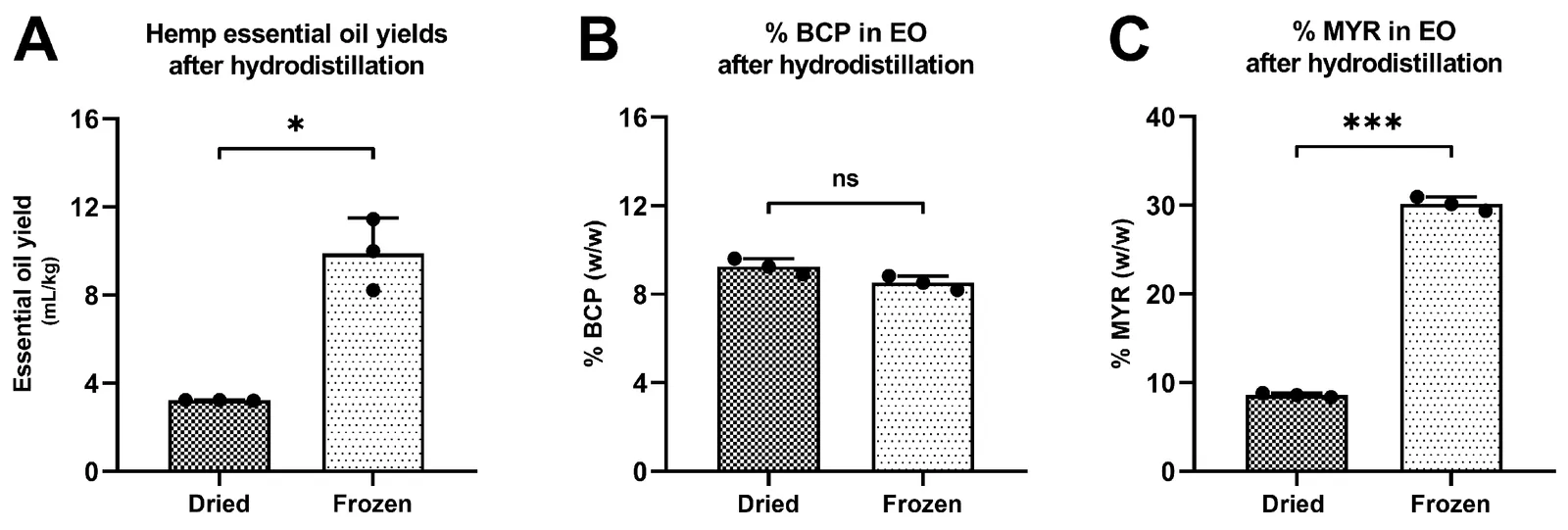
Freezing increases essential oil (EO) yield and preserves the volatile monoterpenes without affecting the sesquiterpenes. Hydrodistillation of frozen (−20 °C) or dried hemp flowers (35 °C), compared for (**A**) essential oil yield (mL/kg), (**B**) β-caryophyllene (BCP) content and (**C**) myrcene (MYR) content (both % *w*/*w* in the essential oil, fraction A). Data are means ± SD (n = 3 independent experiments); unpaired *t*-test with Welch’s correction (ns, not significant; * *p* < 0.05; *** *p* < 0.001).

**Figure 6:**
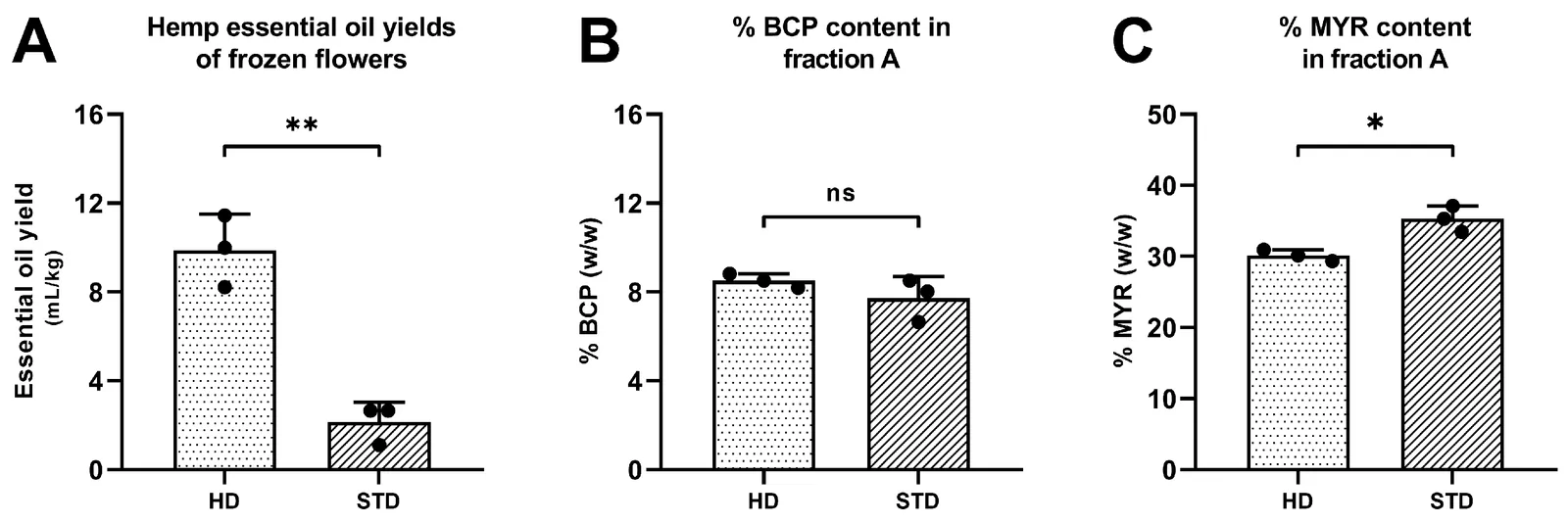
Hydrodistillation (HD) gives a higher essential oil yield than steam distillation (STD). Frozen hemp flowers were processed by HD or STD and compared for (**A**) essential oil yield (mL/kg), (**B**) β-caryophyllene (BCP) content, and (**C**) myrcene (MYR) content (both % *w*/*w* in the essential oil, fraction A). Data are mean ± SD (n = 3 independent experiments); unpaired *t*-test with Welch’s correction (ns, not significant; * *p* < 0.05; ** *p* < 0.01).

**Figure 7:**
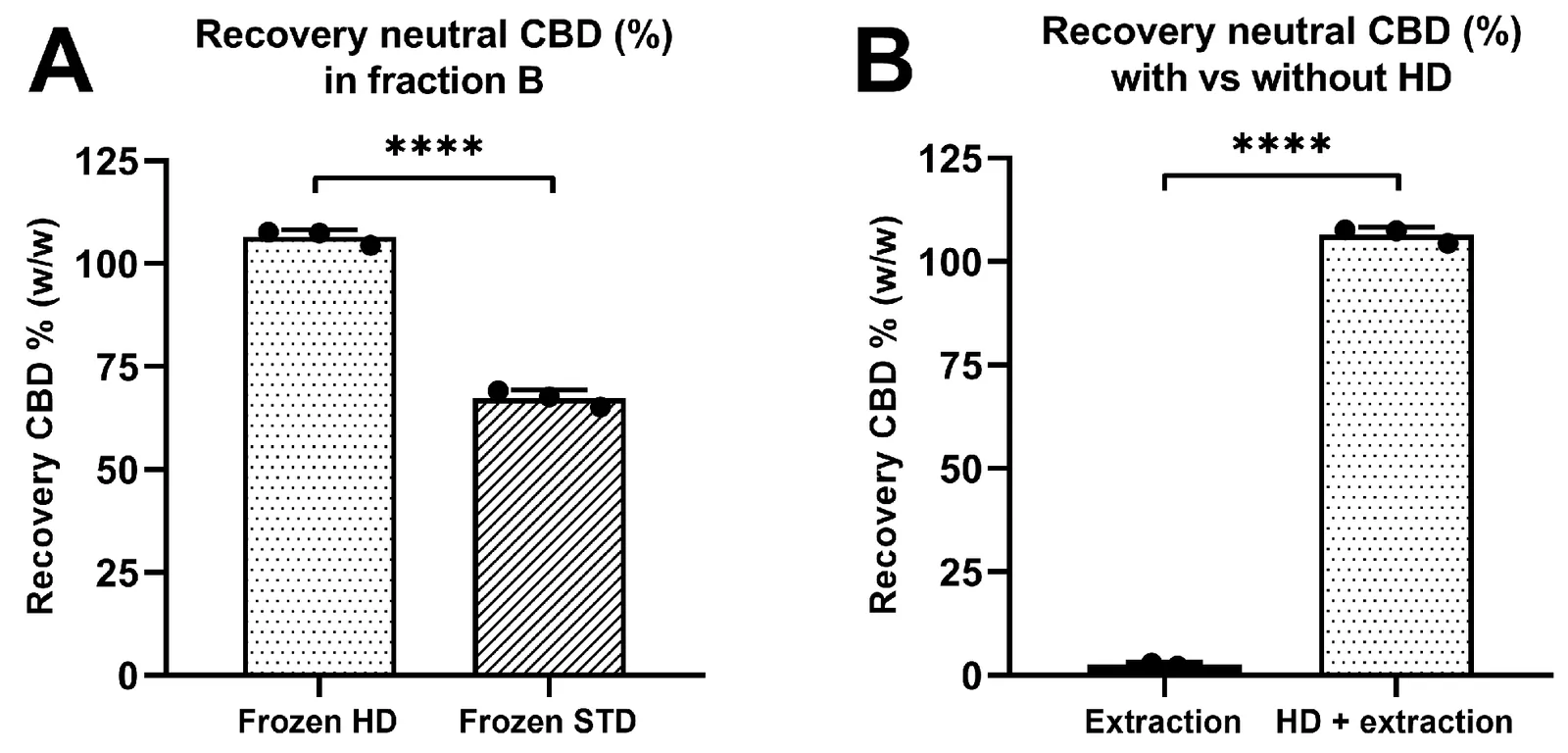
In situ decarboxylation during HD enables complete neutral CBD recovery. Neutral CBD recovery (% *w*/*w*, relative to the total CBD content of the raw material) in the cannabinoid fraction (fraction B). (**A**) Frozen flowers processed by HD versus STD. (**B**) Direct ethanol extraction alone vs. HD followed by extraction (HD + extraction). Data are mean ± SD (n = 3 independent experiments); unpaired *t*-test with Welch’s correction (**** *p* < 0.0001).

**Figure 8:**
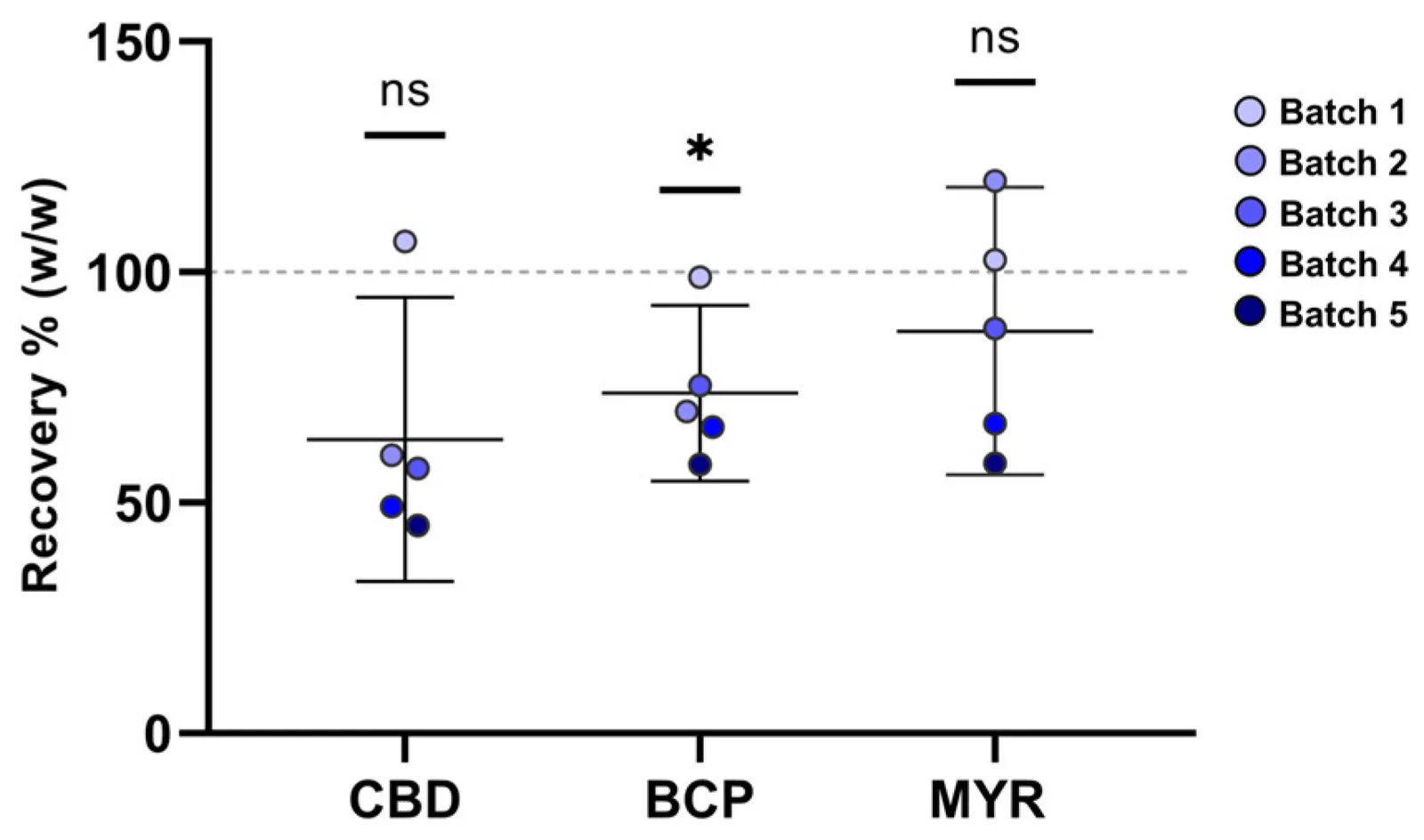
Recovery rates of the three target markers after the complete production process. Recovery (% *w*/*w*) of CBD (cannabinoid marker, HPLC-DAD), β-caryophyllene (BCP, sesquiterpene marker, GC-FID), and myrcene (MYR, monoterpene marker, GC-FID). Each point corresponds to an independent production batch (n = 5); error bars represent the 95% confidence interval of the mean. The dotted line marks 100% recovery (one-sample *t*-test versus 100%; ns = not significant; * *p* = 0.0204 for BCP).

Method development for this integrated process was conducted on Felina 32. Santhica 27 was also tested, but its essential oil yield was too low to justify the full set of optimization experiments (1.47 mL/kg for frozen material). Extraction of the residue obtained after hydrodistillation nonetheless showed that CBGA had been decarboxylated, with the extract containing 7.44% neutral CBG and a recovery of 71.6% relative to the total CBG content of the raw material. For this reason, Figure 5, Figure 6, Figure 7 and Figure 8 focus on Felina 32, on which the integrated process was fully developed and validated.

We first evaluated the terpene fraction. Freezing the biomass rather than low-temperature oven-drying it nearly tripled the essential oil yield obtained by hydrodistillation (3.2 mL/kg for dried versus 9.9 mL/kg for frozen flowers, 95% CI [2.66, 10.68]) and increased its MYR content more than three-fold (8.6% versus 30.2% *w*/*w*, 95% CI [19.80, 23.35]), while the BCP content was unaffected (9.2% versus 8.5%, ns) (Figure 5A–C). This indicates that the volatile monoterpene fraction, rather than the heavier sesquiterpenes, is preferentially lost during oven drying, and that freezing effectively prevents this loss.

Comparing distillation techniques on frozen flowers, HD gave a markedly higher essential oil yield than steam distillation (STD) (9.9 versus 2.1 mL/kg, 95% CI [4.42, 11.06]) and a comparable BCP content (8.5% versus 7.7%, ns), whereas STD gave a slightly higher myrcene content (35.3% versus 30.2% *w*/*w*, 95% CI [1.32, 8.95]) (Figure 6A–C). HD of frozen material was therefore selected as the standard distillation step, on the basis of its higher yield, despite a slightly lower myrcene enrichment relative to STD.

Beyond the two markers quantified against external calibration curves (BCP and MYR, Figure 6), the full terpene profile of the essential oils obtained by HD and STD was characterized by GC-FID and expressed as relative peak area (normalization). This corresponds to the proportion of each compound’s peak area relative to the sum of all terpene peak areas identified in the chromatogram, excluding peaks corresponding to cannabinoids. Identified compounds were confirmed by injection of authentic reference standards. This normalization provides an overview of the overall chemical profile of the essential oil, showing the relative contribution of each terpene to the total composition (Table 1). Overall, the same compounds were identified across both distillation techniques, at similar relative proportions, with the notable exception: α-pinene and β-pinene content was approximately twofold higher under HD than STD, while terpinolene showed the opposite pattern, approximately twofold higher under STD. Monoterpenes accounted for 71.7% and 75.8% of the total terpene profile in HD and STD, respectively. A total of 91.7% (HD) and 92.0% (STD) of the chromatographic peak area was assigned to identified compounds. Representative chromatograms are provided in the Supplementary Materials (Figures S1 and S2).

The residue remaining after hydrodistillation (or steam distillation) was dried overnight in a ventilated oven at 25 °C, reaching a final moisture content of maximum 8%, and extracted using PLE with ethanol. The heat applied during the preceding HD step was sufficient by itself to drive in situ decarboxylation of CBDA and CBGA within the biomass. Extracts prepared from the hydrodistilled residue showed complete decarboxylation of CBD, reaching 106.5% recovery relative to the total CBD content of the raw material, compared with only 67.3% recovery when the residue had instead been steam-distilled (95% CI of the difference [34.9, 43.5]). By contrast, a close-to-zero recovery (2.6%) was observed for neutral CBD when the frozen material was extracted directly with ethanol, without any prior distillation step (95% CI of the difference [99.9, 107.9]), as decarboxylation did not occur (Figure 7A,B).

HD therefore serves a dual purpose in the integrated process: it recovers the terpene fraction as an essential oil while simultaneously supplying the heat required for cannabinoid decarboxylation, decoupling this step entirely from the drying stage. The final cannabinoid extract for Felina 32 gave an extraction yield of 15.52% and contained 22.65 ± 0.32% (*w*/*w*) neutral CBD, with a small residual amount of CBG (0.48 ± 0.05% *w*/*w*). The extract obtained from Santhica 27 after hydrodistillation contained 7.44 ± 0.17% (*w*/*w*) neutral CBG, with a residual CBD content of 0.38 ± 0.04% (*w*/*w*), from an extraction yield of 18.66%. Representative chromatogram of Felina 32 extract obtained by HPLC-DAD is provided in the Supplementary Materials (Figure S3).

To assess the reproducibility and stability of the integrated process over time, the complete workflow (freezing, HD and PLE) was applied to five independent production batches of Felina 32 from the 2024 harvest, processed at intervals of 3 to 6 months over the course of one year. Recovery of the three target markers, CBD, BCP and MYR, was quantified for each batch and compared against a hypothetical value of 100% using a one-sample *t*-test (Figure 8).

Average recovery reached 95.9% for CBD (95% CI [82.2, 109.6]) and 87.8% for MYR (95% CI [55.5, 120.1]), with both intervals including 100%, indicating consistent and near-complete recovery of both cannabinoids and monoterpenes across batches and over time. BCP recovery was lower, averaging 74.3% (95% CI [55.1, 93.5]), an interval that does not include 100%. The wide confidence interval observed for MYR recovery reflects batch-to-batch variability in essential oil yield, which ranged from 6.8 to 11.4 mL/kg across the five batches. Since MYR recovery is calculated as the product of its concentration in the essential oil and the essential oil yield, higher yields directly translate into higher calculated recoveries, accounting for the value of 120% observed in one batch.

### 2.4. Validation of the Integrated Process with an Independent Variety and Harvest Season

To test whether the integrated process also applies beyond Felina 32, the workflow was applied the following year (2025 harvest) to Autopower, a high-CBD variety (≤0.3% THC). The major part of the harvest was frozen to reproduce the process developed in the previous section. As in previous harvests, a representative subsample of Autopower flowers was oven-dried to characterize the raw material, revealing a total extractable CBD content of 7.6% (*w*/*w* DW) and a fresh-to-dry weight loss of 74.06%.

**Figure 9:**
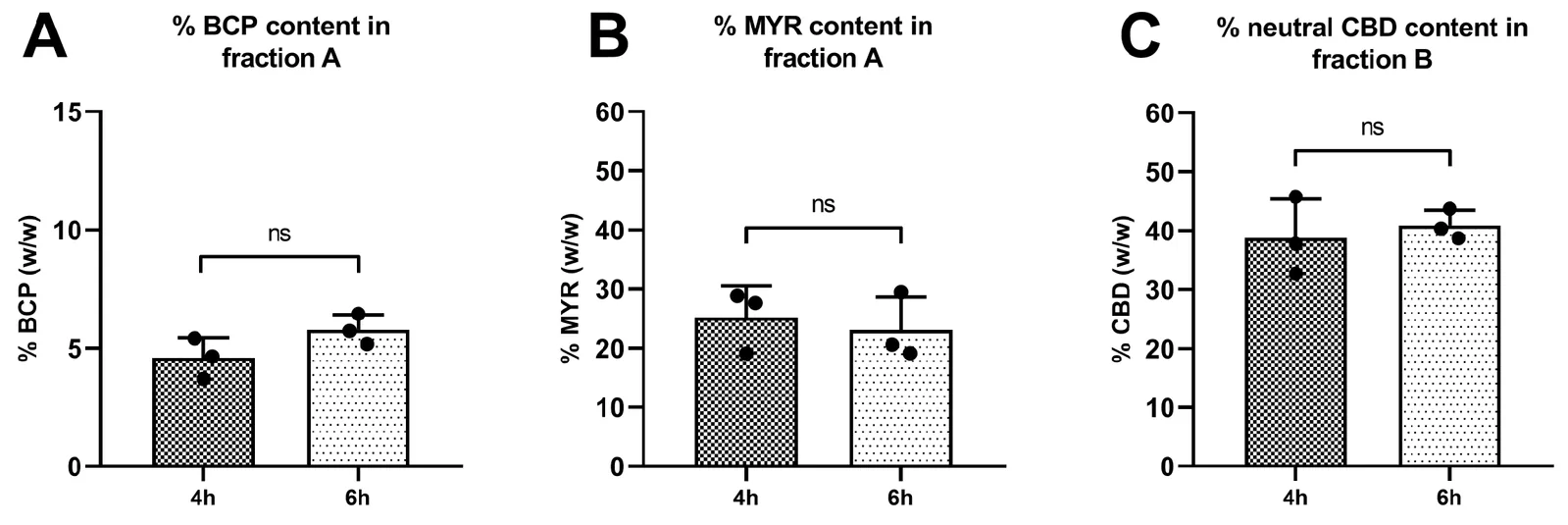
Effect of HD duration on terpene and cannabinoid content in Autopower hemp variety. HD was performed for 4 h or 6 h, and extracts were compared for (**A**) β-caryophyllene (BCP) content and (**B**) myrcene (MYR) content in the essential oil (fraction A) and (**C**) neutral CBD content in the cannabinoid fraction (fraction B). Data are mean ± SD (n = 3 independent experiments); unpaired *t*-test with Welch’s correction (ns, not significant).

In addition to testing the integrated process on a more concentrated variety, we also wanted to verify certain process parameters, including hydrodistillation duration. The effect of hydrodistillation duration was evaluated on Autopower frozen flowers, comparing 4 h and 6 h. Essential oil yield reached 2.33 mL/kg at 4 h and 2.37 mL/kg at 6 h, and subsequent ethanolic extraction yield reached 13.51% and 15.56%, respectively. Extending HD from 4 to 6 h did not significantly affect BCP content either (4.6% versus 5.8% *w*/*w*, ns), nor MYR content (25.2% versus 23.1% *w*/*w*), nor neutral CBD content in the ethanolic fraction (38.8% versus 40.9% *w*/*w*) (Figure 9A–C). As none of these differences reached statistical significance, a 4 h HD can be considered as sufficient to achieve optimal recovery of both terpenes and cannabinoids, consistent with the duration selected for Felina 32. The final cannabinoid extract for Autopower contained 38.8 ± 6.62% (*w*/*w*) neutral CBD, with a residual CBG content of 1.89 ± 0.42% (*w*/*w*) and a crude extraction yield of 13.51%. A representative chromatogram of Autopower extract obtained by HPLC-DAD is provided in the Supplementary Materials (Figure S4).

For the terpene profile, the same major compounds were identified in Autopower flowers, but in different relative proportions (Table 2), determined using the same GC-FID method and reference standards as for Felina 32. Only 77.4% of the total peak area was assigned to identified compounds, compared with over 91% for Felina 32 (Table 1), a larger unidentified fraction that may include additional, uncharacterized terpenes or terpenoid derivatives specific to this variety. This essential oil contained less BCP (6.74% versus 11.60% in Felina 32) and less myrcene (24.43% versus 32.89% in Felina 32), but substantially more p-cymene and terpinolene, along with a notable proportion of bisabolol, which was only a minor constituent in Felina 32. Representative chromatograms are provided in Supplementary Materials (Figure S5). Autopower essential oil was also noted to have a distinctly different odor from Felina 32. The relatively high proportion of p-cymene in Autopower essential oil, nearly absent in Felina 32, may contribute to its distinct spicy, woody odor, although a formal sensory analysis would be needed to confirm this.

## 3. Discussion

Rigorous control of the raw material is a precondition for reproducible outcomes throughout the extraction process of natural products [21]. Raw material composition varies between growing seasons and locations, so characterizing it is what makes studies comparable. It also allows recovery to be calculated, which is the share of the compound present in the raw material that ends up in the final extract. A recovery below 100% means the extraction can still be improved, by running it longer, adding cycles, or using a different solvent, but only if the raw material was quantified first. Without that reference point, there is no way to know whether a process is operating close to its true potential.

The DoE results show that drying temperature is the dominant driver of both CBD/CBG decarboxylation and terpene loss, with no oven-drying condition tested capable of achieving both objectives simultaneously. The four reduced models showed good predictive ability, with R^2^ PRESS values ranging from 0.82 to 0.88 as estimated by leave-one-out cross-validation, indicating that the models explained a substantial proportion of the variance in cannabinoid recovery and extraction yield (Appendix A, Table A2).

Rather than seeking a compromise drying temperature, the process developed here removes the constraint altogether by decoupling terpene recovery and cannabinoid decarboxylation in time and by fraction: terpenes are recovered first, under appropriate conditions (freezing, then hydrodistillation), and decarboxylation is achieved subsequently, driven by the heat of hydrodistillation itself.

Freezing preserved essential oil yield and monoterpene content relative to oven-drying, while sesquiterpene (BCP) content was unaffected by either treatment. Our HPLC analyses of the raw material before and after distillation showed no deterioration or loss of the major cannabinoid peaks, suggesting that distillation at 100 °C for 4 h did not substantially alter the cannabinoid profile nor the recovery. This does not, however, rule out the formation of minor degradation products not targeted by our analytical method.

Zheljazkov and Maggi [20] reported, based on scanning electron microscopy, that most glandular trichomes in distilled hemp biomass remained structurally intact after distillation. This observation concerns the physical integrity of the trichome structure, not the chemical stability of the cannabinoids it contains, which remains governed by the thermal conditions applied. The core principle of the process nonetheless holds: hydrodistillation drives in situ decarboxylation while recovering the terpene fraction, allowing both to be obtained from a single batch of biomass. Minor cannabinoid degradation, however, cannot be entirely excluded.

Freezing also offers a practical advantage for production: essential oil producers typically need to distill fresh material shortly after harvest, whereas frozen biomass can be stored and processed later. This allows several production batches to be spread out over the year rather than processed all at once. In the present study, the five independent production batches were processed at intervals ranging from 1 week to 11 months after harvest, with comparable recoveries observed across all batches (Figure 8), suggesting that storage at −20 °C preserves phytochemical integrity for at least 11 months. Stability beyond this timeframe was not assessed and warrants further investigation.

The full terpene profile obtained for Felina 32 essential oil, dominated by myrcene, terpinolene, α-pinene and β-caryophyllene, is broadly consistent with previous characterizations of cannabis essential oils [22,23,24]. Direct comparison of relative abundances across studies should, however, be made with caution: when hydrodistillation is performed on dried, rather than fresh or frozen, plant material, substantial losses of volatile monoterpenes inflate the relative contribution of the less volatile sesquiterpenes, without this reflecting a genuine difference in composition. This is illustrated by Pieracci et al. [24], who used the same Clevenger-type apparatus as in the present study and reported an essential oil yield of 0.12% (*v*/*w*) from Felina 32 air-dried at room temperature in the dark. This is nearly 10-fold lower than the yield obtained here from frozen flowers of the same variety (mean yield of 0.99% *v*/*w*), reinforcing the advantage of distilling frozen flowers directly. By contrast, Bertoli et al. [23], who hydrodistilled fresh material (from Felina 34 hemp variety) right after harvest, reported a terpene profile dominated by monoterpenes rather than sesquiterpenes, corroborating our own finding that monoterpenes accounted for more than 70% of the relative terpene profile when starting material was not dried before distillation.

Between the two distillation techniques tested on frozen material, hydrodistillation outperformed steam distillation on essential oil yield, while steam distillation gave a modestly higher relative enrichment in some monoterpenes. The lower essential oil yield obtained by steam distillation may reflect uneven vapor penetration through the frozen biomass, resulting in less homogeneous distillation than hydrodistillation, where the plant material is directly immersed in boiling water. The cannabinoid extract obtained after HD and PLE can be used as-is or further purified. If a higher-purity cannabinoid fraction is desired, winterization could be applied as an additional step to remove co-extracted waxes and lipids [25]. All experiments in this study were conducted at laboratory scale. Scaling up the process, particularly HD and PLE, to pilot or industrial volumes remains to be demonstrated, and may affect yield, decarboxylation efficiency, or terpene composition in ways not captured here.

Maximizing recovery of both fractions is not merely a processing goal: cannabinoids and terpenes each carry documented biological activity, and their combination is of pharmacological interest across multiple conditions, from mood and anxiety disorders [26] to cancer [27,28]. BCP, in particular, has been reported to act synergistically with cannabidiol in a pain and inflammation model [29], while myrcene has shown anti-inflammatory and antinociceptive properties in preclinical models [30].

In this respect, a companion study from our group screened close to 250 combinations of CBD, CBG, CBDA, and BCP against triple-negative breast cancer cells, evaluated using SynergyFinder. This screen provides a catalogue of highly synergistic combinations [31]. Recovering both fractions efficiently and reproducibly is therefore a prerequisite for exploiting this biological potential, rather than an end in itself.

## 4. Materials and Methods

### 4.1. Plant Material

Female hemp inflorescences (THC ≤ 0.3% *w*/*w*, in accordance with EU regulation) were cultivated in an open field in Wallonia, Belgium, and harvested at maturity in early autumn. Four batches were used across the study: a preliminary batch harvested in September 2022, and three subsequent batches comprising Felina 32 (CBD-dominant) and Santhica 27 (CBG-dominant), harvested in September 2023 and September 2024, and Auto power (high-CBD, THC ≤ 0.3% *w*/*w*), harvested in October 2025. Detailed sourcing information is provided in Appendix A (Table A1). For each harvest, stems and leaves were removed to retain only the inflorescences, which were then either frozen directly at −20 °C or dried in a ventilated oven (Memmert GmbH & Co. KG, Schwabach, Germany) at the required temperature for 24–72 h, depending on the experiment.

### 4.2. Design of Experiment (DoE): Impact of Drying Conditions on Cannabinoid Recovery

This study aimed to evaluate the influence of the drying temperature on cannabinoid profiles in the extracts. A D-optimal experimental design was implemented using JMP Pro 15 (SAS Institute, Cary, NC, USA) to assess the main effects, first-order interactions, and quadratic effects of three continuous factors (drying temperature, extraction temperature, and duration of each extraction cycle) and one discrete numeric factor (number of extraction cycles, at three levels: 3, 4, or 5). Drying temperature was tested at three levels (35, 50, and 70 °C). The design comprised 24 individual runs (Table 3), applied to both hemp varieties, Felina 32 and Santhica 27. Each experimental condition was extracted once per variety, with phytochemical quantification performed in triplicate. Two responses were monitored: extraction yield and the recovery of neutral CBD (Felina 32) or neutral CBG (Santhica 27).

For each response, a reduced model was derived from the full model by retaining the most significant effects, interactions and quadratic terms, together with the main effects required by model hierarchy. Model quality (R^2^ and RMSE) was assessed by leave-one-out cross-validation (PRESS; Appendix A, Table A2). Predicted responses were visualized by Monte Carlo simulation (5000 runs) of the reduced models: the two most influential factors were varied across their tested range, the remaining factors were drawn uniformly within their ranges, and residual noise was added according to the model RMSE.

### 4.3. Analytical Method: HPLC (CBD/CBDA, CBG/CBGA) and GC (β-Caryophyllene, Myrcene)

#### 4.3.1. High-Performance Liquid Chromatography (HPLC)

Cannabinoids were analyzed by HPLC-DAD according to the validated method of De Backer et al. [32], with minor adaptations. The mobile phase consisted of a mixture of methanol/water containing 50 mM ammonium formate (adjusted to pH 5.2 with formic acid). The initial condition was 68% methanol (*v*/*v*), linearly increased to 90.5% methanol over 25 min, then to 95% over 1 min. This condition was maintained for 3 min, after which the column was returned to initial conditions over 1 min and re-equilibrated for 6 min (total runtime: 36 min). The flow rate was 0.6 mL/min, the injection volume was 10 µL, and the column temperature was maintained at 30 °C. Detection was performed at 225 nm. Samples were prepared by weighing 200.0 mg of raw material or 20.0 mg of extract into a final volume of 10.0 mL of methanol. Methanol (5 mL) was first added, followed by 10 min of ultrasonic bath treatment, brief vortexing, and a further 10 min in the ultrasonic bath. The volume was then completed to 10.0 mL with methanol, and the sample solution was filtered through a 0.45 µm filter.

Calibration standard solutions of CBD were prepared in methanol over the range 0.015625–0.5 mg/mL. Reference standards for CBDA, CBGA, CBD, and CBG were purchased from PhytoLab (Bavaria, Germany). A single calibration curve of CBD was used for quantification, and CBDA, CBG, and CBGA were quantified using their respective relative response factors to CBD, determined experimentally: 0.488 for CBDA, 0.985 for CBG, and 0.560 for CBGA. Total recoverable CBD in the plant material was calculated as the sum of the neutral CBD content (% *w*/*w*) and the CBDA content, the latter converted to its neutral equivalent using a molecular weight correction factor of 0.877 (MW CBD/MW CBDA). The same approach was applied to CBG and CBGA, using a molecular weight correction factor of 0.877.

The analytical column used was a Waters XTerra RP18 (5 µM, 250 mm, 4.6 mm), protected by a Waters XTerra RP18 VanGuard cartridge (5 µm, 5 mm, 3.9 mm). All runs were carried out using an HPLC 1290 Infinity II system (Agilent Technologies, Waldbronn, Germany), consisting of a G7104A quaternary solvent pump, a G7129B autosampler, a G7130A column oven and a G7117D DAD (diode-array detector). Equipment control and data acquisition were performed with Agilent OpenLab CDS Chemstation C.01.10 (Agilent Technologies, Waldbronn, Germany).

#### 4.3.2. Gas Chromatography (GC)

Volatile compounds in hemp raw material and extracts were analyzed on an Agilent Intuvo 9000 (G3950A) gas chromatograph (Agilent Technologies, Waldbronn, Germany) coupled to a flame-ionization detector (FID), according to Pellati et al. [33], with a slightly modified temperature gradient. Compounds were separated on an HP-5MS UI capillary column (30 m × 0.32 mm, 0.25 µm). The column temperature was initially set at 45 °C, increased to 100 °C at 2 °C/min, then to 270 °C at 5 °C/min, and held for 5 min (total runtime: 66.5 min). Helium was used as carrier gas at a flow rate of 1.0 mL/min. Samples were injected directly (injection volume: 1 µL, split ratio 10:1). Injector and detector temperatures were set at 250 °C and 300 °C, respectively. Calibration solutions of β-caryophyllene (0.010–0.25 mg/mL) and myrcene (0.0025–0.4000 mg/mL) were prepared in hexane and used to quantify the most abundant sesquiterpene and monoterpene, respectively. Thymol was used as an internal standard at a concentration of 0.04 mg/mL; 20 µL of internal standard solution was added to each sample (final volume: 1000 µL).

Raw material samples were prepared by extracting 200 mg of dried, ground flowers in 3 mL of hexane (10 min ultrasonic bath), or 1 g of frozen flowers, ground directly in hexane using a dispersing instrument (IKA Ultra-Turrax T8, Staufen, Germany), followed by 10 min in an ultrasonic bath. Essential oil samples were prepared at a concentration of 0.75 mg/mL in hexane. All hemp volatile compounds were identified by comparison of retention times with those of authentic reference standards analyzed under the same conditions. BCP and MYR were quantified against their respective calibration curves, and the remaining identified terpenes were expressed as relative percentages of the total terpene profile.

### 4.4. Yield and Recovery Calculation

All results are expressed relative to the dry weight (DW) of the starting material. For batches processed as frozen biomass, a fresh-to-dry weight ratio was determined for each harvest by oven-drying a representative subsample, allowing all subsequent yields and recoveries to be expressed on a common dry weight basis, regardless of whether the biomass was dried or frozen prior to processing.

Recovery of each cannabinoid was calculated as follows:

CBD recovery (%) = ([CBD]extract × extraction yield)/[CBD]raw × 100

where [CBD]extract is the neutral CBD content of the extract (% *w*/*w*) and [CBD]raw is the total CBD content of the raw material (CBD + CBDA, expressed as CBD equivalents, % *w*/*w*). The same approach was applied to CBG (CBG + CBGA, expressed as CBG equivalents). For terpenes, recovery was calculated analogously, using the essential oil yield in place of the extraction yield, and the total terpene content of the raw material as the denominator.

### 4.5. Distillation: Hemp Essential Oil

Two techniques were used to extract terpenes in this study. Hydrodistillation was performed using the apparatus for essential oil determination described in the European Pharmacopoeia (Clevenger-type apparatus, Ph. Eur. 2.8.12), consisting of a 1 L round-bottom flask and a heating mantle. In the flask, 500 mL of purified water was introduced together with 40 g of plant material (dried or frozen, not grinded). Distillation was carried out for 4 to 6 h. Essential oil was then collected, residual water was removed, and the oil was stored at −20 °C. Steam distillation was also used to compare the phytochemical profile and extraction yield with those obtained by hydrodistillation. Steam distillation was performed using a “Nano” stainless steel distillation extractor (AlChemia Solutions, Saguenay, QC, Canada). The grid was raised using aluminum spacers to accommodate smaller amounts of plant material. The stainless-steel tank was filled with water to a level 5 cm below the grid supporting the plant material. Distillation was carried out for 4 h.

### 4.6. Ethanolic Extraction of Cannabinoids

Extraction was performed using an automated system for pressurized liquid extraction system (E-914, SpeedExtractor, Büchi, Flawil, Switzerland), at 100 bar. Extractions were carried out using 5 g of ground plant material and 25–30 g of 0.3–0.9 mm quartz sand (Büchi, Flawil, Switzerland) to fill the 40 mL extraction cell, with ethanol (Disinfectol^®^, ref. CL00.0112, AnalytiChem Belgium NV, Zedelgem, Belgium). For the Design of Experiments, extractions were performed according to the temperature, number of cycles, and cycle duration indicated in the DoE (Section 4.2). For the integrated process, the reference extraction method consisted of 4 cycles of 37 min at 30 °C. Each cycle was followed by 6 min of discharge and 1 min of heating before the next cycle. Extracts were then evaporated using a rotary evaporator and then stored at −20 °C.

### 4.7. Statistical Analysis

The influence of each parameter on cannabinoid recovery and extraction yield was evaluated using a D-optimal Design of Experiments, generated and analyzed in JMP Pro 15 (SAS Institute, Cary, NC, USA), as described in Section 4.2. Pairwise comparisons between two experimental conditions were performed using an unpaired two-tailed Welch’s *t*-test; 95% confidence intervals of the mean difference are reported where relevant. Recovery values expressed relative to a fixed reference (100% recovery, Figure 8) were analyzed by one-sample *t*-test against a theoretical mean of 100, with 95% confidence intervals of the mean reported. Unless otherwise stated, data are presented as mean ± SD from at least three independent experiments, and differences were considered statistically significant at *p* < 0.05 (ns, not significant; * *p* < 0.05, ** *p* < 0.01, *** *p* < 0.001, **** *p* < 0.0001). Pairwise and one-sample *t*-tests, along with the corresponding graphs, were performed using GraphPad Prism 8 (GraphPad Software, Boston, MA, USA).

## 5. Conclusions

This study resolves an apparent conflict in cannabis processing: initiating cannabinoid decarboxylation has traditionally come at the cost of terpene loss, since both have been treated as outcomes of a single thermal step. By decoupling them in time and by fraction, freezing to preserve terpenes, and then hydrodistillation to recover them as an essential oil while supplying the heat needed to decarboxylate the cannabinoids remaining in the residual biomass, this trade-off is resolved without compromising either fraction.

Design of Experiments analysis identified drying temperature as the critical parameter governing decarboxylation under conventional conditions, providing the rationale for this redesign. This work shows that reproducibility in plant extraction depends on control exercised across the entire workflow, not on extraction optimization alone. The resulting process recovers both an essential oil and a cannabinoid fraction from a single batch of biomass, offering a route toward broad-spectrum, tailored-composition hemp products.

## Figures and Tables

**Table 1:** Relative terpene composition (% of total identified terpene peak area) of essential oils obtained by hydrodistillation (HD) and steam distillation (STD) of frozen Felina 32 flowers, determined by GC-FID. Data are mean ± SD (n = 3 independent experiments).

RT (min)	Compound	Relative Area (%) HD	Relative Area (%) STD
12.300	α-Pinene	11.62 ± 0.86	5.08 ± 0.08
13.031	Camphene	0.11 ± 0.013	0.10 ± 0.00
14.514	β-Pinene	4.08 ± 0.16	2.62 ± 0.02
15.405	Myrcene	33.02 ± 0.77	32.89 ± 0.30
16.066	α-Phellandrene	0.33 ± 0.13	0.68 ± 0.00
16.399	3-Carene	0.53 ± 0.04	0.56 ± 0.00
16.805	α-Terpinene	-	0.52 ± 0.00
17.252	p-Cymene	0.52 ± 0.03	-
17.505	Limonene	1.65 ± 0.22	1.98 ± 0.01
18.099	cis-ocimene	0.56 ± 0.14	0.55 ± 0.00
18.739	trans-ocimene	7.08 ± 1.38	8.53 ± 0.08
21.214	Terpinolene	12.10 ± 2.73	22.08 ± 0.09
21.975	Linalool	0.13 ± 0.11	0.23 ± 0.19
37.487	β-caryophyllene	11.60 ± 0.06	9.65 ± 0.10
38.497	Humulene	5.44 ± 0.23	4.81 ± 0.10
39.665	Valencene	0.42 ± 0.11	0.56 ± 0.01
41.363	Nerolidol	0.65 ± 0.18	0.65 ± 0.18
41.995	Caryophyllene oxide	1.26 ± 0.90	0.53 ± 0.43
44.253	Bisabolol	0.42 ± 0.11	-
	Total identified %	91.70	92.02

**Table 2:** Relative terpene composition (% of total identified terpene peak area) of essential oils obtained by hydrodistillation (HD) of frozen Autopower flowers, determined by GC-FID. Data are mean ± SD (n = 3 independent experiments).

RT (min)	Compound	Relative Area (%) HD
12.300	α-Pinene	2.81 ± 0.06
13.031	Camphene	0.13 ± 0.01
14.514	β-Pinene	2.62 ± 0.04
15.405	Myrcene	24.43 ± 0.32
16.066	α-Phellandrene	0.67 ± 0.01
16.399	3-Carene	0.53 ± 0.01
16.805	α-Terpinene	0.60 ± 0.02
17.252	p-Cymene	7.25 ± 0.08
17.505	Limonene	-
18.099	cis-ocimene	0.18 ± 0.02
18.739	trans-ocimene	3.32 ± 0.03
21.214	Terpinolene	18.92 ± 0.12
21.975	Linalool	0.23 ± 0.02
27.913	α-terpineol	0.93 ± 0.01
37.487	β-caryophyllene	6.74 ± 0.13
38.497	Humulene	2.62 ± 0.05
39.665	Valencene	0.31 ± 0.01
41.363	Nerolidol	0.71 ± 0.01
41.995	Caryophyllene oxide	0.44 ± 0.02
44.253	Bisabolol	2.69 ± 0.08
	Total identified %	77.40

**Table 3:** D-Optimal experimental plan created with JMP Pro 15 program for evaluation of drying conditions on cannabinoid recovery after extraction.

Run	Drying Temperature (°C)	Extraction Temperature (°C)	Cycle Duration (min)	Number of Cycles
1	70	100	15	5
2	70	65	38	4
3	35	65	60	5
4	70	30	15	3
5	50	30	60	4
6	50	65	38	4
7	35	30	38	5
8	70	65	38	4
9	35	100	15	3
10	70	30	60	3
11	35	100	60	4
12	70	100	60	5
13	70	30	15	5
14	35	30	15	4
15	35	100	60	3
16	50	65	15	5
17	35	30	38	3
18	50	100	40	5
19	35	57	15	3
20	70	30	60	5
21	70	100	60	3
22	35	30	60	3
23	70	100	15	3
24	35	100	15	5

## Data Availability

The original contributions presented in this study are included in the article and its Supplementary Materials. Further inquiries can be directed to the corresponding author, M.F.

## Supplementary Materials

The following supporting information can be downloaded at https://www.mdpi.com/article/10.3390/molecules31213955/s1, Table S1: Individual results of the 24-run D-optimal Design of Experiments performed on Felina 32 variety; Table S2: Individual results of the 24-run D-optimal Design of Experiments performed on Santhica 27 variety; Table S3: Analysis of Variance, Lack of Fit, and Parameter Estimates for the reduced DoE model of extraction yield (%) in the Felina 32 variety; Table S4: Analysis of Variance, Lack of Fit, and Parameter Estimates for the reduced DoE model of extraction yield (%) in the Santhica 27 variety; Table S5: Analysis of Variance, Lack of Fit, and Parameter Estimates for the reduced DoE model of CBD recovery (%) in the Felina 32 variety; Table S6: Analysis of Variance, Lack of Fit, and Parameter Estimates for the reduced DoE model of CBG recovery (%) in the Santhica 27 variety; Figure S1: Representative GC-FID chromatogram of the essential oil fraction from frozen Felina 32 flowers (obtained by hydrodistillation). Thymol was used as internal standard for quantification. Figure S2: Representative GC-FID chromatogram of the essential oil fraction from frozen Felina 32 flowers (obtained by steam distillation). Thymol was used as internal standard for quantification. Figure S3: Representative HPLC-DAD chromatogram of the cannabinoid extract obtained after hydrodistillation (HD) of frozen Felina 32 flowers. Figure S4: Representative HPLC-DAD chromatogram of the cannabinoid extract obtained after hydrodistillation (HD) of frozen Autopower flowers. Figure S5: Representative GC-FID chromatogram of the essential oil fraction from frozen Autopower flowers (obtained by hydrodistillation). Thymol was used as internal standard for quantification.

## Abbreviations

The following abbreviations are used in this manuscript:

BCP	β-caryophyllene
CBD	Cannabidiol
CBDA	Cannabidiolic acid
CBG	Cannabigerol
CBGA	Cannabigerolic acid
DW	Dry weight
DoE	Design of Experiments
GC-FID	Gas Chromatography with Flame Ionization Detection
HD	Hydrodistillation
HPLC-DAD	High-Performance Liquid Chromatography with Diode Array Detection
MYR	Myrcene
PLE	Pressurized Liquid Extraction
PRESS	Predicted Residual Sum of Squares
RMSE	Root Mean Square Error
SD	Standard Deviation
STD	Steam Distillation
THC	Tetrahydrocannabinol
THCA	Tetrahydrocannabinolic acid

## Appendix A

**Table A1:** Sourcing and harvest details of the hemp batches used in this study.

Batch	Year	Harvest Date	Variety	Source
1	2022	16/08/2022 25/08/2022	Santhica 27 Felina 32	Integrated and Urban Plant Pathology Laboratory, Gembloux Agro-Bio Tech
2	2023	31/08/2023 15/09/2023	Santhica 27 Felina 32	Integrated and Urban Plant Pathology Laboratory, Gembloux Agro-Bio Tech
3	2024	23/09/2024 23/09/2024	Santhica 27 Felina 32	CBX Medical SRL, Charleroi, Belgium
4	2025	01/10/2025	Autopower	Cannavie, Walhain, Belgium

**Table A2:** Terms retained in the reduced model for each response, with model fit statistics obtained by leave-one-out cross-validation (PRESS).

Response	Variety	Terms Retained	R^2^ (PRESS)	RMSE (PRESS)
CBD recovery	Felina 32	Drying temperature, extraction temperature, drying temperature^2^, cycle duration × cycles, drying temperature × extraction temperature, cycles *, cycle duration *	0.880	11.305
CBG recovery	Santhica 27	Drying temperature, drying temperature^2^, cycle duration × cycles, extraction temperature, drying temperature × cycles, cycles *, cycle duration *	0.881	7.199
Extraction yield	Felina 32	Extraction temperature, drying temperature, drying temperature × extraction temperature, drying temperature^2^, cycles	0.822	1.216
Extraction yield	Santhica 27	Extraction temperature, drying temperature^2^, cycle duration, cycles^2^, cycles *, drying temperature *	0.822	1.627

Terms marked with an asterisk were retained to respect model hierarchy (main effects involved in a retained interaction or quadratic term), regardless of their individual significance.

## References

[1] Tomko A.M., Whynot E.G., Ellis L.D., Dupré D.J. (2020). Anti-Cancer Potential of Cannabinoids, Terpenes, and Flavonoids Present in Cannabis. Cancers.

[2] Andre C.M., Hausman J.-F., Guerriero G. (2016). *Cannabis sativa*: The Plant of the Thousand and One Molecules. Front. Plant Sci..

[3] Ben-Shabat S., Fride E., Sheskin T., Tamiri T., Rhee M.-H., Vogel Z., Bisogno T., De Petrocellis L., Di Marzo V., Mechoulam R. (1998). An Entourage Effect: Inactive Endogenous Fatty Acid Glycerol Esters Enhance 2-Arachidonoyl-Glycerol Cannabinoid Activity. Eur. J. Pharmacol..

[4] Russo E.B. (2011). Taming THC: Potential Cannabis Synergy and Phytocannabinoid-Terpenoid Entourage Effects. Br. J. Pharmacol..

[5] Chacon F.T., Raup-Konsavage W.M., Vrana K.E., Kellogg J.J. (2022). Secondary Terpenes in *Cannabis sativa* L.: Synthesis and Synergy. Biomedicines.

[6] Anand U., Pacchetti B., Anand P., Sodergren M.H. (2021). Cannabis-Based Medicines and Pain: A Review of Potential Synergistic and Entourage Effects. Pain Manag..

[7] Blasco-Benito S., Seijo-Vila M., Caro-Villalobos M., Tundidor I., Andradas C., García-Taboada E., Wade J., Smith S., Guzmán M., Pérez-Gómez E. (2018). Appraising the “Entourage Effect”: Antitumor Action of a Pure Cannabinoid versus a Botanical Drug Preparation in Preclinical Models of Breast Cancer. Biochem. Pharmacol..

[8] Christensen C., Rose M., Cornett C., Allesø M. (2023). Decoding the Postulated Entourage Effect of Medicinal Cannabis: What It Is and What It Isn’t. Biomedicines.

[9] André R., Gomes A.P., Pereira-Leite C., Marques-da-Costa A., Monteiro Rodrigues L., Sassano M., Rijo P., Costa M.d.C. (2024). The Entourage Effect in Cannabis Medicinal Products: A Comprehensive Review. Pharmaceuticals.

[10] Da Costa-Oliveira C., Gouvêa-Silva J.G., dos Santos Fernandes Muniz R., Felisberto J.S., Mazzola P.G., Pereira R.C., de Lima Moreira D., Ramos Y.J. (2026). Cannabis: From Crop to Shop—Some Insights about Stability to Access Quality Control. J. Cannabis Res..

[11] Addo P.W., Desaulniers Brousseau V., Morello V., MacPherson S., Paris M., Lefsrud M. (2021). Cannabis Chemistry, Post-Harvest Processing Methods and Secondary Metabolite Profiling: A Review. Ind. Crops Prod..

[12] Das P.C., Vista A.R., Tabil L.G., Baik O.-D. (2022). Postharvest Operations of Cannabis and Their Effect on Cannabinoid Content: A Review. Bioengineering.

[13] Lazarjani M.P., Young O., Kebede L., Seyfoddin A. (2021). Processing and Extraction Methods of Medicinal Cannabis: A Narrative Review. J. Cannabis Res..

[14] AL Ubeed H.M.S., Wills R.B.H., Chandrapala J. (2022). Post-Harvest Operations to Generate High-Quality Medicinal Cannabis Products: A Systemic Review. Molecules.

[15] Baek Y., Grab H., Chen C. (2025). Postharvest Drying and Curing Affect Cannabinoid Contents and Microbial Levels in Industrial Hemp (*Cannabis sativa* L.). Plants.

[16] Cvetanov A., Bogevski K., Maksimova V., Ivanovska E.J. (2026). Post-harvest drying of *Cannabis sativa* L. flowers and its impact on the preservation and extraction of bioactive phytochemicals. J. Agric. Plant Sci..

[17] European Directorate for the Quality of Medicines & HealthCare (EDQM) (2023). Cannabis Flower European Pharmacopoeia.

[18] Chacon F.T., Raup-Konsavage W.M., Vrana K.E., Kellogg J.J. (2024). Effect of Hemp Extraction Procedures on Cannabinoid and Terpenoid Composition. Plants.

[19] Gunjević V., Grillo G., Carnaroglio D., Binello A., Barge A., Cravotto G. (2021). Selective Recovery of Terpenes, Polyphenols and Cannabinoids from *Cannabis sativa* L. Inflorescences under Microwaves. Ind. Crops Prod..

[20] Zheljazkov V.D., Maggi F. (2021). Valorization of CBD-Hemp through Distillation to Provide Essential Oil and Improved Cannabinoids Profile. Sci. Rep..

[21] Wang H., Chen Y., Wang L., Liu Q., Yang S., Wang C. (2023). Advancing Herbal Medicine: Enhancing Product Quality and Safety through Robust Quality Control Practices. Front. Pharmacol..

[22] Micalizzi G., Alibrando F., Vento F., Trovato E., Zoccali M., Guarnaccia P., Dugo P., Mondello L. (2021). Development of a Novel Microwave Distillation Technique for the Isolation of *Cannabis sativa* L. Essential Oil and Gas Chromatography Analyses for the Comprehensive Characterization of Terpenes and Terpenoids, Including Their Enantio-Distribution. Molecules.

[23] Bertoli A., Tozzi S., Pistelli L., Angelini L.G. (2010). Fibre Hemp Inflorescences: From Crop-Residues to Essential Oil Production. Ind. Crops Prod..

[24] Pieracci Y., Ascrizzi R., Terreni V., Pistelli L., Flamini G., Bassolino L., Fulvio F., Montanari M., Paris R. (2021). Essential Oil of *Cannabis sativa* L: Comparison of Yield and Chemical Composition of 11 Hemp Genotypes. Molecules.

[25] Haczkiewicz M., Świtalska M., Łyczko J., Wietrzyk J., Gliszczyńska A. (2025). Phytochemical Profile, Extraction and Characterization of Bioactive Compounds from Industrial Hemp (*Cannabis sativa* L.) Felina 32 Variety. Molecules.

[26] Ferber S.G., Namdar D., Hen-Shoval D., Eger G., Koltai H., Shoval G., Shbiro L., Weller A. (2020). The “Entourage Effect”: Terpenes Coupled with Cannabinoids for the Treatment of Mood Disorders and Anxiety Disorders. Curr. Neuropharmacol..

[27] Tomko A.M., Whynot E.G., O’Leary L.F., Dupré D.J. (2022). Anti-Cancer Potential of Cannabis Terpenes in a Taxol-Resistant Model of Breast Cancer. Can. J. Physiol. Pharmacol..

[28] Aare M., Lazarte J.M., Nathani A., Boirie B., Turley T.N., Copland J.A., Singh M. (2026). Synergistic Anticancer Activity of Cannabinoids and Terpenes Against Triple-Negative Breast Cancer Resistance. Int. J. Mol. Sci..

[29] Blanton H., Yin L., Duong J., Benamar K. (2022). Cannabidiol and Beta-Caryophyllene in Combination: A Therapeutic Functional Interaction. Int. J. Mol. Sci..

[30] McDougall J.J., McKenna M.K. (2022). Anti-Inflammatory and Analgesic Properties of the Cannabis Terpene Myrcene in Rat Adjuvant Monoarthritis. Int. J. Mol. Sci..

[31] Hamann C., Jansen O., Jullien K., Lhonneux L., Ledoux A., Frédérich M., Maquoi E. (2026). Synergistic Induction of a Lethal Autosis-to-Apoptosis Switch by Phytocannabinoids and Beta-Caryophyllene in Triple-Negative Breast Cancer Cells. bioRxiv.

[32] De Backer B., Debrus B., Lebrun P., Theunis L., Dubois N., Decock L., Verstraete A., Hubert P., Charlier C. (2009). Innovative Development and Validation of an HPLC/DAD Method for the Qualitative and Quantitative Determination of Major Cannabinoids in Cannabis Plant Material. J. Chromatogr. B.

[33] Pellati F., Brighenti V., Sperlea J., Marchetti L., Bertelli D., Benvenuti S. (2018). New Methods for the Comprehensive Analysis of Bioactive Compounds in *Cannabis sativa* L. (Hemp). Molecules.

