# Supplementary material for: From Field to Extract: Controlling Post-Harvest and Extraction Variables for Reproducible Recovery of Cannabinoids and Terpenes from Cannabis sativa L. by Sequential Hydrodistillation and Pressurized Liquid Extraction
Source: Molecules. 2026 Nov 2;31(21):3955. doi: 10.3390/molecules31213955 (PMC13765217; doi:10.3390/molecules31213955)

Table S1: Individual results of the 24-run D-optimal Design of Experiments performed on Felina 32 variety.

N°	Drying temperature (°C)	Extraction temperature (°C)	Duration of extraction cycle (min)	Number of cycles	Yield of extraction (%)	% neutral CBD (w/w)	Recovery neutral CBD (%)	% BCP (w/w)
**1**	70	100	15	5	**28.25**	4.62 ± 0.54	104.20 ± 9.98	0.03
**2**	70	65	38	4	**24.94**	5.00 ± 0.26	99.86 ± 6.81	0.03
**3**	35	65	60	5	**26.64**	1.46 ± 0.06	23.48 ± 1.62	0.30
**4**	70	30	15	3	**18.38**	5.91 ± 0.58	87.01 ± 9.62	0.03
**5**	50	30	60	4	**22.87**	3.42 ± 0.08	37.12 ± 1.66	0.18
**6**	50	65	38	4	**26.24**	3.17 ± 0.30	39.55 ± 3.87	0.16
**7**	35	30	38	5	**22.35**	1.48 ± 0.11	19.97 ± 1.93	0.26
**8**	70	65	38	4	**21.89**	5.67 ± 0.19	99.32 ± 5.44	0.03
**9**	35	100	15	3	**26.73**	1.50 ± 0.32	24.08 ± 5.27	0.26
**10**	70	30	60	3	**19.94**	5.29 ± 0.34	84.33 ± 4.98	0.03
**11**	35	100	60	4	**27.35**	2.87 ± 0.20	47.26 ± 4.62	0.12
**12**	70	100	60	5	**26.48**	4.52 ± 0.57	95.71 ± 12.08	0.03
**13**	70	30	15	5	**19.10**	6.12 ± 0.33	93.51 ± 5.82	0.03
**14**	35	30	15	4	**21.72**	1.45 ± 0.13	19.04 ± 2.25	0.13
**15**	35	100	60	3	**25.53**	4.07 ± 0.08	62.63 ± 0.61	0.12
**16**	50	65	15	5	**25.35**	5.10 ± 0.39	61.38 ± 4.66	0.07
**17**	35	30	38	3	**23.19**	1.56 ± 0.11	21.75 ± 2.09	0.15
**18**	50	100	40	5	**28.42**	4.03 ± 0.43	54.40 ± 6.60	0.06
**19**	35	57	15	3	**24.48**	1.46 ± 0.14	21.56 ± 2.61	0.14
**20**	70	30	60	5	**22.06**	5.73 ± 0.41	101.10 ± 8.24	0.03
**21**	70	100	60	3	**26.59**	4.89 ± 0.07	103.87 ± 2.99	0.02
**22**	35	30	60	3	**20.76**	1.38 ± 0.12	17.23 ± 1.93	0.14
**23**	70	100	15	3	**26.21**	4.71 ± 0.16	98.68 ± 4.18	0.02
**24**	35	100	15	5	**26.97**	4.29 ± 0.27	69.71 ± 6.13	0.13

Table S2: Individual results of the 24-run D-optimal Design of Experiments performed on Santhica 27 variety.

N°	Drying temperature (°C)	Extraction temperature (°C)	Duration of extraction cycle (min)	Number of cycles	Yield of extraction (%)	% neutral CBG (w/w)	Recovery neutral CBG (%)	% BCP (w/w)
**1**	70	100	15	5	**18.38**	5.18 ± 0.35	83.47 ± 3.96	0.04
**2**	70	65	38	4	**13.90**	5.04 ± 0.11	61.49 ± 2.81	0.04
**3**	35	65	60	5	**12.67**	1.64 ± 0.18	14.60 ± 1.07	0.19
**4**	70	30	15	3	**7.00**	7.71 ± 0.18	47.40 ± 2.32	0.04
**5**	50	30	60	4	**17.13**	1.48 ± 0.12	16.70 ± 1.92	0.12
**6**	50	65	38	4	**17.07**	1.27 ± 0.10	14.19 ± 1.66	0.10
**7**	35	30	38	5	**11.24**	1.93 ± 0.18	15.25 ± 1.16	0.25
**8**	70	65	38	4	**13.93**	4.93 ± 0.28	60.38 ± 4.77	0.04
**9**	35	100	15	3	**16.39**	1.80 ± 0.06	20.82 ± 0.58	0.23
**10**	70	30	60	3	**9.57**	7.02 ± 0.17	58.96 ± 0.48	0.04
**11**	35	100	60	4	**19.51**	2.69 ± 0.23	37.02 ± 2.91	0.16
**12**	70	100	60	5	**18.69**	4.19 ± 0.08	68.76 ± 2.96	0.03
**13**	70	30	15	5	**8.69**	7.79 ± 0.48	59.44 ± 4.34	0.05
**14**	35	30	15	4	**10.24**	2.27 ± 0.12	16.36 ± 0.23	0.18
**15**	35	100	60	3	**15.92**	4.12 ± 0.05	46.23 ± 1.40	0.16
**16**	50	65	15	5	**15.45**	3.74 ± 0.10	37.87 ± 2.30	0.12
**17**	35	30	38	3	**10.57**	2.74 ± 0.07	20.41 ± 0.45	0.19
**18**	50	100	40	5	**21.35**	1.80 ± 0.15	25.27 ±2.90	0.10
**19**	35	57	15	3	**12.55**	2.15 ± 0.09	19.03 ± 0.47	0.21
**20**	70	30	60	5	**10.00**	6.88 ± 0.31	60.44 ± 3.87	0.04
**21**	70	100	60	3	**18.38**	4.05 ± 0.32	65.39 ± 6.67	0.02
**22**	35	30	60	3	**10.69**	2.89 ± 0.07	21.80 ± 0.42	0.14
**23**	70	100	15	3	**13.44**	3.97 ± 0.09	46.86 ± 2.04	0.02
**24**	35	100	15	5	**18.62**	2.05 ± 0.12	26.97 ± 1.59	0.14

Table S3. Analysis of Variance, Lack of Fit, and Parameter Estimates for the reduced DoE model of extraction yield (%) in the Felina 32 variety

.

**Analysis of Variance**					
**Source**	**DF**	**SS**	**MS**	**F**	**p-value**
Model	5	177.971	35.594	30.439	<0.0001
Error	18	21.048	1.169		
C. Total	23	199.020			
**Lack of Fit**					
**Source**	**DF**	**SS**	**MS**	**F**	**p-value**
Lack of Fit	11	5.489	0.499	0.225	0.9860
Pure Error	7	15.560	2.223		
Total Error	18	21.048			
**Parameter Estimates**					
**Term**	**Estimate (coded)**	**Std Error**	**t Ratio**	**p-value**	**Estimate (uncoded)**
Intercept	25.401	0.571	44.52	<0.0001	11.343
Drying temperature	-0.670	0.243	-2.75	0.0131	0.359
Drying temperature × Drying temp.	-1.347	0.628	-2.14	0.0459	-0.004
Extraction temperature	2.870	0.255	11.25	<0.0001	0.030
Cycles	0.501	0.266	1.88	0.0759	0.501
Drying temp. × Extraction temp.	0.609	0.270	2.26	0.0367	0.001

DF: Degree of Freedom, SS: Sum of squares, MS: Mean square, F: F ratio

Table S4. Analysis of Variance, Lack of Fit, and Parameter Estimates for the reduced DoE model of extraction yield (%) in the Santhica 27 variety.

**Analysis of Variance**					
**Source**	**DF**	**SS**	**MS**	**F**	**p-value**
Model	6	325.612	54.269	29.294	<0.0001
Error	17	31.494	1.853		
C. Total	23	357.106			
**Lack of Fit**					
**Source**	**DF**	**SS**	**MS**	**F**	**p-value**
Lack of Fit	16	31.493	1.968	4374.1	0.0119
Pure Error	1	0.000	0.000		
Total Error	17	31.493			
**Parameter Estimates**					
**Term**	**Estimate (coded)**	**Std Error**	**t Ratio**	**p-value**	**Estimate (uncoded)**
Intercept	18.071	0.788	22.94	<0.0001	-42.484
Drying temperature	-0.423	0.306	-1.38	0.1852	1.155
Drying temperature × Drying temp.	-3.438	0.820	-4.19	0.0006	-0.011
Extraction temperature	3.707	0.323	11.48	<0.0001	0.106
Cycle duration	0.778	0.323	2.41	0.0277	0.035
Cycles	0.595	0.336	1.77	0.0945	10.755
Cycles × Cycles	-1.270	0.673	-1.89	0.0765	-1.270

DF: Degree of Freedom, SS: Sum of squares, MS: Mean square, F: F ratio

Table S5. Analysis of Variance, Lack of Fit, and Parameter Estimates for the reduced DoE model of CBD recovery (%) in the Felina 32 variety.

**Analysis of Variance**					
**Source**	**DF**	**SS**	**MS**	**F**	**p-value**
Model	7	24464.057	3494.865	47.924	<0.0001
Error	16	1166.810	72.926		
C. Total	23	25630.866			
**Lack of Fit**					
**Source**	**DF**	**SS**	**MS**	**F**	**p-value**
Lack of Fit	15	1166.630	77.775	432.085	0.0377
Pure Error	1	0.180	0.180		
Total Error	16	1166.810			
**Parameter Estimates**					
**Term**	**Estimate (coded)**	**Std Error**	**t Ratio**	**p-value**	**Estimate (uncoded)**
Intercept	48.601	4.532	10.72	<0.0001	0.855
Drying temperature	31.324	1.921	16.30	<0.0001	-3.372
Drying temperature × Drying temp.	16.785	4.984	3.37	0.0039	0.055
Extraction temperature	9.803	2.017	4.86	0.0002	0.759
Cycle duration	-0.130	2.020	-0.06	0.9493	1.123
Cycles	3.350	2.105	1.59	0.1311	13.930
Drying temp. × Extraction temp.	-5.588	2.130	-2.62	0.0184	-0.009
Cycle duration × Cycles	-6.348	2.224	-2.85	0.0115	-0.282

DF: Degree of Freedom, SS: Sum of squares, MS: Mean square, F: F ratio

Table S6. Analysis of Variance, Lack of Fit, and Parameter Estimates for the reduced DoE model of CBG recovery (%) in the Santhica 27 variety.

**Analysis of Variance**					
**Source**	**DF**	**SS**	**MS**	**F**	**p-value**
Model	7	9959.273	1422.753	45.294	<0.0001
Error	16	502.586	31.412		
C. Total	23	10461.860			
**Lack of Fit**					
**Source**	**DF**	**SS**	**MS**	**F**	**p-value**
Lack of Fit	15	501.986	33.466	55.315	0.1052
Pure Error	1	0.605	0.605		
Total Error	16	502.586			
**Parameter Estimates**					
**Term**	**Estimate (coded)**	**Std Error**	**t Ratio**	**p-value**	**Estimate (uncoded)**
Intercept	23.507	2.978	7.89	<0.0001	129.150
Drying temperature	18.883	1.269	14.88	<0.0001	-6.487
Drying temperature × Drying temp.	18.823	3.283	5.73	<0.0001	0.061
Extraction temperature	5.774	1.324	4.36	0.0005	0.165
Cycle duration	1.703	1.326	1.28	0.2173	1.224
Cycles	2.226	1.381	1.61	0.1267	-1.612
Drying temperature × Cycles	4.866	1.419	3.43	0.0034	0.278
Cycle duration × Cycles	-6.457	1.459	-4.42	0.0004	-0.287

DF: Degree of Freedom, SS: Sum of squares, MS: Mean square, F: F ratio

Figure S1: Representative GC-FID chromatogram of the essential oil fraction from frozen Felina 32 flowers (obtained by hydrodistillation). Thymol was used as internal standard for quantification.

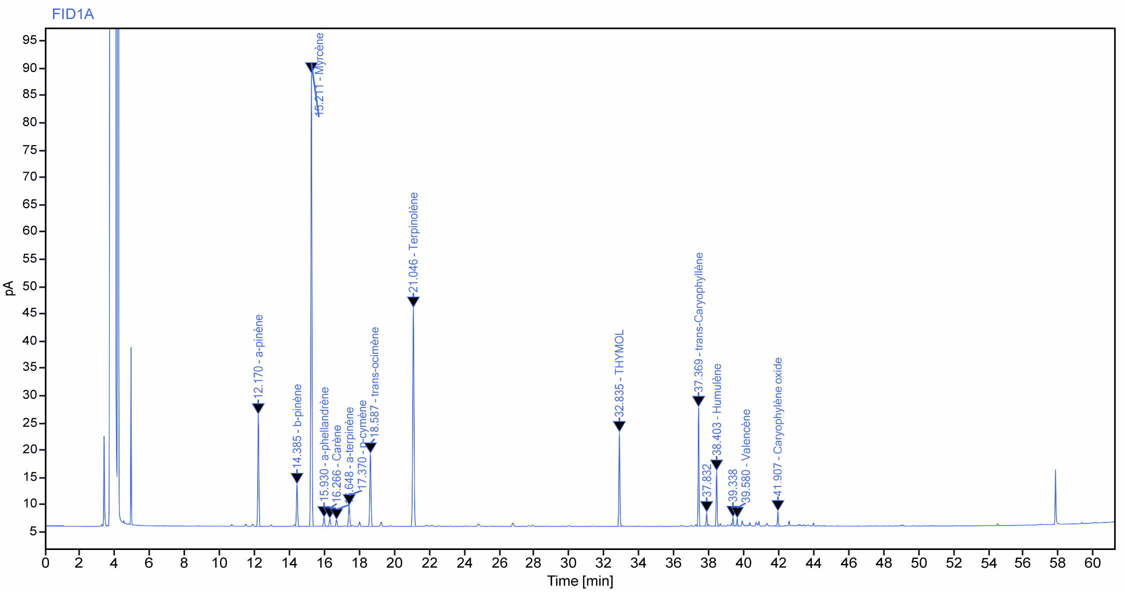

Figure S2: Representative GC-FID chromatogram of the essential oil fraction from frozen Felina 32 flowers (obtained by steam distillation). Thymol was used as internal standard for quantification.

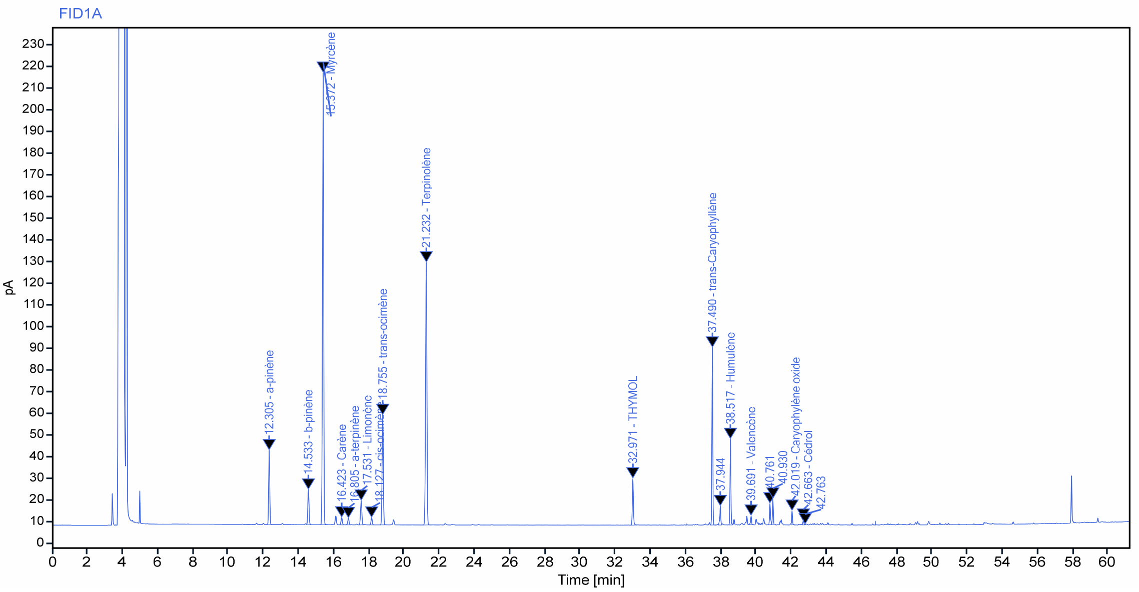

Figure S3: Representative HPLC-DAD chromatogram of the cannabinoid extract obtained after hydrodistillation (HD) of frozen Felina 32 flowers.

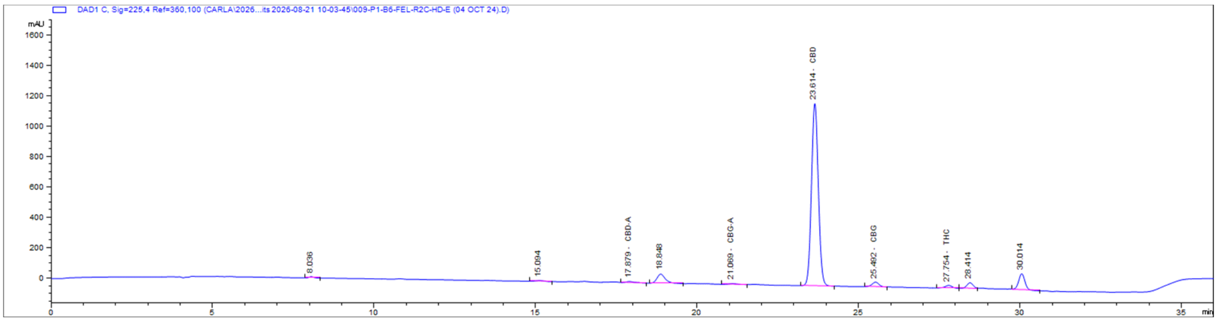

Figure S4: Representative HPLC-DAD chromatogram of the cannabinoid extract obtained after hydrodistillation (HD) of frozen Autopower flowers.

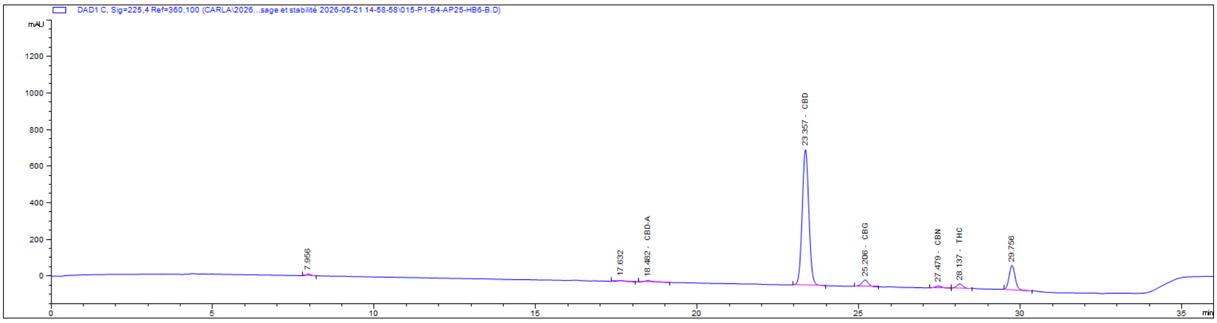

Figure S5: Representative GC-FID chromatogram of the essential oil fraction from frozen Autopower flowers (obtained by hydrodistillation). Thymol was used as internal standard for quantification.

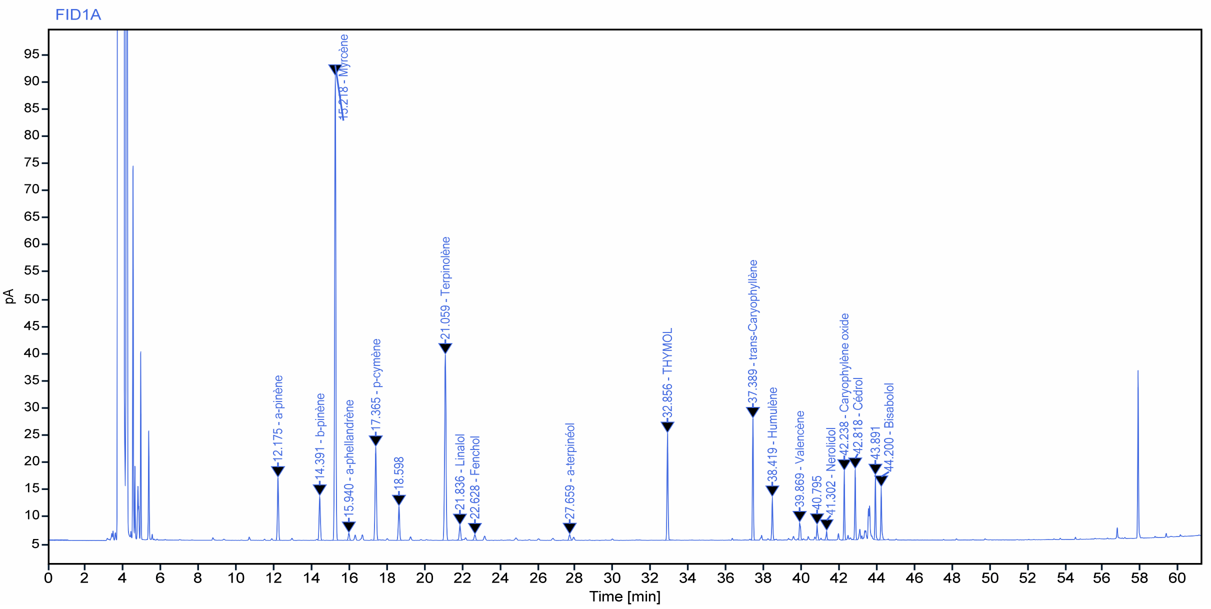

Supplement: Supplementary file 1 [file molecules-31-03955-s001.zip › molecules-4641560-supplementary.pdf]
